# High MHC diversity confers no advantage for phenotypic quality and reproductive performance in a wild bird

**DOI:** 10.1111/1365-2656.13737

**Published:** 2022-05-15

**Authors:** Ewa Pikus, Peter O. Dunn, Piotr Minias

**Affiliations:** ^1^ Department of Biodiversity Studies and Bioeducation, Faculty of Biology and Environmental Protection University of Łódź Łódź Poland; ^2^ Behavioral and Molecular Ecology Group, Department of Biological Sciences University of Wisconsin‐Milwaukee Milwaukee WI USA

**Keywords:** birds, fitness, heterozygote advantage, major histocompatibility complex, MHC diversity, optimality hypothesis

## Abstract

1. Genes of the major histocompatibility complex (MHC) encode antigen‐binding molecules and are an integral part of the acquired immune response of vertebrates. In general, high individual MHC diversity is expected to increase fitness by broadening the spectrum of pathogens recognized by the immune system, in accordance with the heterozygote advantage mechanism. On the other hand, the optimality hypothesis assumes that individuals with optimal (intermediate), rather than maximum, diversity of the MHC will achieve the highest fitness because of inherent costs associated with expressing diverse MHC alleles.
2. Here, we tested for associations between individual diversity of the MHC class I and class II genes (binding antigens of intra‐ and extracellular pathogens respectively) and a range of fitness‐related traits (condition, ornament expression and reproduction) in an urban population of the Eurasian coot *Fulica atra*.
3. Contrary to our expectation, we found that high within‐individual allelic diversity of MHC genes (both class I and II) was associated with poorer condition (lower blood haemoglobin concentrations), weaker expression of the putative ornament (smaller frontal shield), later onset of breeding and smaller clutches. An analysis of functional MHC allele clusters (supertypes) provided further support for negative associations of MHC diversity with phenotypic quality and reproductive performance, but most of these relationships could not be explained by the presence of specific maladaptive supertypes. Finally, we found little empirical support for the optimality hypothesis in the Eurasian coot.
4. Our results suggest that the costs of high MHC diversity outweighed any benefits associated with broad MHC repertoire, which could be driven by depauperate pathogen diversity in an urban landscape. To the best of our knowledge, this is one of the first studies providing consistent evidence for negative associations of MHC diversity with a range of fitness‐related traits in a natural avian population.

## Graphical abstract

A long‐term monitoring of an urban population of the Eurasian coot shows that high allelic diversity of the major histocompatibility complex (MHC) genes is associated with poorer condition, weaker ornamental expression, and lower reproductive parameters. Thus, it provides evidence for an unfavourable cost‐benefit ratio of diverse MHC repertoire.

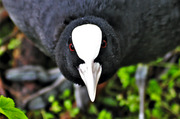

## 1 INTRODUCTION

The family of major histocompatibility complex (MHC) genes forms the most polymorphic region within the vertebrate genome and it plays a critical role in the response of the acquired immune system (Geraghty et al., 2002). MHC molecules bind fragments of peptides derived from the processing of intracellular (MHC class I) or extracellular (MHC class II) pathogens and display them for recognition by the appropriate T lymphocytes, which initiates the production of antibodies or the destruction of pathogen‐infected cells (Janeway Jr et al., 2001). As a result of the co‐evolutionary arms race between pathogens and their hosts, the antigen‐binding domains of MHC molecules are subjected to strong diversifying and balancing selection, which leads to the maintenance of high diversity of these genes in natural populations (Spurgin & Richardson, 2010).

There are several non‐exclusive mechanisms of balancing selection that promote the maintenance of high MHC diversity within natural populations and these processes can be further reinforced by sexual selection (Ejsmond et al., 2014; Spurgin & Richardson, 2010). In general, females may prefer to mate with males that have ‘good genes’, that is alleles that can directly improve the genetic quality of their offspring (Brouwer et al., 2010; Dunn et al., 2013). This kind of mating mechanism may be explained by the rare allele hypothesis (negative frequency‐dependent selection), where individuals with low‐frequency MHC alleles gain a fitness advantage, as their immune system may better detect and counteract pathogens with novel mutations that avoid the most common MHC alleles of the host (Brouwer et al., 2010; Gillingham et al., 2017). Another hypothesis proposes that the maximum diversity of alleles at the individual level may be the most favourable in terms of fitness. According to the heterozygote advantage mechanism (overdominant selection), a greater number of alleles expressed within an individual increases the spectrum of antigens recognized and, thus, provides a selective advantage in pathogen recognition, thereby improving fitness (Doherty & Zinkernagel, 1975). Consistently, MHC genes of various vertebrate lineages have been subject to extensive duplication processes and duplicated gene copies have often been retained by selection processes and conserved throughout their evolution, which leads to an increased diversity of MHC alleles (Axtner & Sommer, 2007). Although it is usually difficult to separate the mechanisms responsible for maintaining high MHC diversity at the population level (Spurgin & Richardson, 2010), the mechanism of heterozygote advantage predicts a positive relationship between MHC diversity and fitness‐related traits across individuals (henceforth referred to as the MHC diversity hypothesis).

The mechanisms of heterozygote advantage do not necessarily consider any upper limits on the richness of MHC alleles within individuals. However, high MHC diversity at the individual level may be unfavourable due to an increased risk of autoimmune diseases and a reduction in the repertoire of antigens recognized by T lymphocyte receptors (TCRs; Nowak et al., 1992; Todd et al., 1988). Recent studies on bank voles *Myodes glareolus* confirmed that a reduction of TCR repertoire size is associated with a high diversity of MHC class I genes, but no similar relationship has been demonstrated for MHC class II genes (Migalska et al., 2019). These mechanisms are directly associated with the hypothesis of optimality (Wegner et al., 2003), assuming that intermediate rather than maximum diversity of MHC alleles is optimal and associated with the highest individual fitness.

So far, research into the associations between individual MHC diversity and fitness components in birds has yielded mixed results. On the one hand, positive linear relationships between MHC diversity and reproduction or survival have been demonstrated in species such as the Egyptian vulture *Neophron percnopterus* (Agudo et al., 2012), Magellanic penguin *Spheniscus magellanicus* (Knafler et al., 2012) and common yellowthroat *Geothlypis trichas* (Dunn et al., 2013). Individual MHC diversity was also reported to be positively correlated with the expression of sexual ornaments (Hale et al., 2009; Whittingham et al., 2015) and negatively associated with the prevalence or intensity of infection by different types of parasites (Radwan et al., 2012; Slade et al., 2017). On the other hand, many studies demonstrated a lack of significant relationships (e.g. in the great snipe *Gallinago media* and greater prairie chicken *Tympanuchus cupido*; Bateson et al., 2016; Ekblom et al., 2004), suggesting that the link between the MHC and fitness is not a universal phenomenon in natural bird populations. However, most research has tested for linear relationships between MHC diversity and fitness‐related traits, while attempts to test the assumptions of the optimality hypothesis in birds have been fewer and mostly inconclusive or negative (e.g. Biedrzycka et al., 2018; Radwan et al., 2012). Limited support for MHC optimality in birds clearly contrasts with the results of similar studies in fish (Forsberg et al., 2007; Hablützel et al., 2014), especially three‐spined stickleback *Gasterosteus aculeatus*, which provide one of the most convincing examples for optimal MHC diversity so far (Kalbe et al., 2009; Kurtz et al., 2004; Wegner et al., 2003).

The aim of this study was to test for associations between the diversity of MHC genes and fitness‐related traits (condition, reproduction and ornament expression) in the Eurasian coot *Fulica atra*, a common waterbird. For this purpose, we genotyped MHC class I and class II in over 100 individuals from a recently established urban population in central Poland and, at the same time, collected information on nearly 200 reproductive episodes of genotyped individuals. We tested for both positive linear and nonlinear associations between MHC diversity and fitness‐related traits, as predicted by the MHC diversity and optimality hypotheses respectively. We also expected that the patterns of associations between the MHC and fitness may differ between class I and class II genes, as the costs of expressing a large number of MHC alleles may vary between both classes (Migalska et al., 2019).

## 2 MATERIALS AND METHODS

### 2.1 Study site and general methodology

Fieldwork took place in an urban area of Łódź (51°45′N, 19°27′E), one of the largest cities in Poland (680,000 inhabitants; 293.25 km^2^). In 2010–2020, we monitored the entire coot population within the administrative borders of the city (*c*. 30–60 breeding pairs per year). During this period, we captured and sampled blood from 114 adult individuals. All birds were captured with noose traps made from monofilament nylon or by hand, while incubating on the nest or while feeding on the shore (at the pre‐laying stage or during the chick rearing period). Each bird was marked with a metal ring (left tarsus) and a plastic neck collar with an individual alphanumerical code that allowed easy identification of individuals in the field. At capture, we took basic measurements, including tarsus length and total head length, both measured with callipers (±0.1 mm). Body mass was measured with an electronic balance (±1 g). We also measured the size of the putative non‐plumage ornament, the frontal shield (see below for details). Finally, we collected ca. 5 μl of blood for the measurement of the total blood haemoglobin concentration (as an indicator of physiological condition, see below), while 50 μl of blood was collected into 96% ethanol for genetic analyses: molecular sexing, MHC genotyping and double digest restriction‐site association DNA (ddRAD) sequencing. Genomic DNA was extracted from all blood samples using GeneJET Genomic DNA Purification Kit (Fermentas, Thermo Fisher Scientific) and Bio‐Trace DNA Purification Kit (EURx), following the manufacturers' protocol. Molecular sexing followed methodology developed by Griffiths et al. (1998), according to the protocol described in Minias (2015a). The sex ratio of our sample was roughly equal (58 females and 56 males). All applicable institutional and/or national guidelines for the care and use of animals were followed during the study and all experiments were conducted by permission of the Local Bioethical Commission for Experiments on Animals in Łódź, Poland (nos. 40/ŁB 620/2012 and 15/ŁB/2016).

### 2.2 Phenotypic traits

We measured three traits (Table 1) that were expected to be associated with phenotypic quality of birds:

- Body mass was used as a general measure of condition, since it may be a reliable indicator of energy reserves, when appropriately corrected for structural body size (Peig & Green, 2009). To estimate structural body size, we calculated the first principal component (PC1) from two size measurements, tarsus and total head length, and included it as a covariate in the modelling.
- Total blood haemoglobin concentration was used as a measure of physiological condition. The measurement was conducted with a portable HemoCue Hb 201+ photometer (HemoCue, Ängeholm, Sweden), which was previously shown to reliably measure this trait in avian blood (Velguth et al., 2010). Absorbance of blood (directly proportional to haemoglobin concentration) was measured using disposable HemoCue microcuvettes. Haemoglobin concentration is a key indicator of blood oxygen‐carrying capacity and it reflects the potential of an organism to satisfy its oxygen demands. In birds, it has been reported to correlate with other measures of condition, diet quality, parasite prevalence and survival across a wide range of taxa (reviewed in Minias, 2015b). Our previous research on coots showed that haemoglobin concentrations were higher in early than late breeding pairs and they strongly varied with urbanization level, thus possibly reflecting variation in physiological condition mediated by changes in diet (Minias, 2016). At the same time, we acknowledge that this measure of condition should be treated with caution until validated more directly in the coot.
- Frontal shield size was used as a measure of a putative ornament expression. Eurasian coots are sexually monochromatic in plumage, but both sexes have a conspicuous and sexually dimorphic (larger in males; Minias, 2015a) white fleshy frontal shield that extends from the bill onto the head crown. This kind of morphological structure is typical for many rallid species and it has been shown in the mooorhen *Gallinula chloropus* (a close relative of the coot) that it increases in size in a testosterone‐dependent manner prior to the breeding season (Eens et al., 2000). Frontal shield size has been reported to signal dominance, social status, fighting ability and condition in other rallids (Alvarez et al., 2005; Dey et al., 2014). In the closely related American coot *Fulica americana*, individuals with large frontal shields were dominant over the ones with smaller shields (Gullion, 1951) and males with smaller shields were more frequently subject to conspecific brood parasitism (Lyon, 2003). Behavioural observations of Eurasian coots confirmed that the frontal shield is a display structure used in competitor assessment (Visser, 1988). We used callipers to measure length and width of the frontal shield (±0.1 mm) and calculated the first principal component (PC1) from these two measurements, which was used as an indicator of ornament size.

### 2.3 Reproductive traits

During the study period, we recorded 206 reproductive events (clutches) by 106 marked individuals (Table 1). For most reproductive events (85.4%), we sampled only one bird per breeding pair. We recorded up to seven reproductive events per individual, but 67% of individuals had only ≤2 reproductive events recorded. Most of the events represented first clutches (82.8%), but there were also renest clutches after brood failure (12.9%) and second clutches (4.3%) included in the dataset. For each reproductive event, laying of the first egg (laying date) was assigned to the successive 5‐day periods beginning from March 20 (earliest laying date recorded within our study population). For most of the events, we also recorded clutch size (*n* = 171), hatching success (coded as hatched versus non‐hatched clutches, *n* = 225) and breeding success (number of fully grown offspring recorded ca. 1.5 month from hatching; *n* = 204) (Table 1). Some reproductive traits were inter‐correlated, for example laying date was negatively associated with clutch size (*p* < 0.001) and breeding success (*p* = 0.009), while larger clutches were associated with higher breeding success (*p* = 0.012).

### 2.4 MHC genotyping

We genotyped the MHC class I and class II gene fragments coding for the peptide‐binding region of the molecule (i.e. the region directly involved in antigen binding). To genotype MHC class I, we used two degenerate primers *MHCI‐int2F* (5′‐ CATTTCCCTYGTGTTTCAGG‐3′) and *MHCI‐ex4R* (3’‐GGGTAGAAGCCGTGAGCRC‐5′) originally designed for accipitrid birds (Alcaide et al., 2009). These two primers bind to the flanking region of intron 2 and the conserved region of exon 4 and successfully amplify almost the entire MHC class I exon 3 (273 bp out of 276 bp). A posteriori verification of primer specificity against MHC class I sequences retrieved from the recently assembled Eurasian coot genome (GenBank number: GCA_013372525.1) showed no mismatches within the 3‐terminal region, which is crucial for effective PCR amplifications (Kwok et al., 1990) and, consequently, non‐specific MHC class I amplifications (allele drop out) were unlikely in our study. For MHC class II genotyping, we used two primers *Fuat‐Ex2Fw* (5′‐CTGACCRGCCTCCCTGCA‐3′) and *Fuat‐Ex2Rv* (5′‐TTGTGCCAYACACCCACC‐3′) originally designed for the Eurasian coot (Alcaide et al., 2014). The primers bind to the flanking region of intron 1 and the conserved region of exon 3 and successfully amplify the entire MHC class II exon 2 (270 bp). All PCR amplifications followed original protocols (Alcaide et al., 2009, 2014). Each PCR sample contained 1 μl of template DNA corresponding to approximately 20–80 ng of DNA, 10 μl of 2X HotStarTaq Plus MasterMix Kit (Qiagen, Venlo, The Netherlands), 8 μl of deionized water and 0.5 μl of each primer (total volume of 20 μl). In all PCRs, we used fusion primers, with Illumina Nextera Transposase adapter sequences (Illumina Corp.), and 7‐bp barcodes for sample identification. We used a relatively low number of amplification cycles (*n* = 25) to reduce the risk of chimera formation. To detect positive amplifications, all PCR products were evaluated by electrophoresis on 2% agarose gel. All PCR products were purified using AMPure XP magnetic beads (Beckman Coulter) and their concentrations were assessed with Quant‐iT PicoGreen dsDNA marking kit (Thermo FisherScientific). The libraries were prepared separately for MHC class I and class II from equimolar quantities of PCR products using NEB‐Next DNA Library Prep Master Mix Set for Illumina (New England Biolabs) and sequenced on the 2 × 250 bp Illumina MiSeq platform.

### 2.5 Processing of Illumina data and MHC allele validation

Raw data from Illumina MiSeq sequencing were analysed and processed using the Amplicon Sequencing Analysis Tools (AmlpiSAT) webserver (Sebastian et al., 2016), following recommendations for Illumina data processing developed by Biedrzycka et al. (2017). The details are presented in the Electronic Supplementary Material (ESM). All validated MHC class I and class II sequences were aligned using Geneious v10.0.0.5 (Biomatters Ltd.). Intron regions were removed from the alignments and alleles were inferred based on the exon fragments only. We retrieved 1–10 MHC class I and 1–6 MHC class II alleles per individual, indicating that our primers targeted at least five and three MHC class I and class II loci, respectively. Although the total number of MHC genes is not known in our study species (as in most non‐model avian taxa), it has been reported to be generally low in other non‐passerine birds (compared to passerines) and only exceptionally exceeds five genes per class (Minias et al., 2019). Individual MHC allelic diversity was calculated as the number of MHC class I or class II alleles recorded per individual, and there was no significant correlation between the number of MHC class I and class II alleles detected per individual (*r* = −0.03, *n* = 114, *p* = 0.76). Nonlinear associations between MHC diversity and fitness‐related traits were tested using both uncategorized raw data (squared term of MHC diversity added as a covariate) or categorized data (three‐level MHC diversity added as a fixed factor). For the purpose of the categorization, MHC diversity was classified as low (1–2 class I or class II alleles), intermediate (3–5 class I alleles or 3 class II alleles) and high (6–10 class I alleles or 4–6 class II alleles).

### 2.6 MHC supertype clustering

As different MHC alleles may have similar peptide‐binding properties and focusing on functional MHC allele clusters (supertypes) may be more biologically relevant (Biedrzycka et al., 2018), we have performed allele clustering for MHC class I and II sequences detected in our study population. For this purpose, we used k‐means clustering and discriminant function analysis of principal components (DAPC) implemented in the adegenet package (Jombart, 2008) developed for the R statistical environment (R Foundation for Statistical Computing, Vienna, Austria). In the analyses, we followed a protocol described by Biedrzycka et al. (2018) and details of allele clustering are presented in the ESM. The MHC supertype diversity was calculated as the number of supertypes detected within an individual. Allele and supertype diversity were significantly correlated at both MHC class I (*r* = 0.80, *n* = 114, *p* < 0.001) and class II (*r* = 0.88, *n* = 114, *p* < 0.001). Frequencies and diversity of MHC supertypes showed no significant temporal trends across the entire study period (all *p* > 0.05). Since the analyses of MHC allelic diversity provided little support for nonlinear associations with fitness‐related traits (see Section 3 for details), we avoided categorization of these data and tested for linear associations only.

### 2.7 ddRAD sequencing

Allelic diversity at the MHC may not only be governed by evolutionary or population processes that specifically target these genes, but may also be affected by the mechanisms operating at the level of the entire genome (e.g. inbreeding). To test whether any possible associations of MHC diversity with fitness were actually driven by genome‐wide diversity, we used ddRAD sequencing to estimate genome‐wide heterozygosity of all captured individuals. The ddRAD libraries were prepared at the Texas A&M AgriLife Genomics Facility (USA), which followed a protocol recommended by Peterson et al. (2012). The details of library preparation and data processing are in the ESM. Genome‐wide heterozygosity was calculated across 14,525 SNPs and ranged from 0.168 to 0.223 (on average 0.203 ± 0.001 [*SE*]). No significant correlation was found between heterozygosity and MHC class I (*r* = 0.03, *n* = 114, *p* = 0.78) or class II (*r* = 0.07, *n* = 114, *p* = 0.43) allele diversity.

### 2.8 Statistical analyses

Associations of MHC class I and class II diversity with fitness‐related (phenotypic and reproductive) traits were analysed using GLMMs, as implemented in the glmmadmb R package (Skaug et al., 2012). In the analyses, we used three different measures of MHC diversity: (i) categorized (ordinal) three‐level MHC allelic diversity; (ii) uncategorized (discrete) MHC allelic diversity; and (iii) uncategorized MHC supertype diversity. All phenotypic (blood haemoglobin concentration, body mass and frontal shield size) and reproductive (laying date, clutch size, hatching success and breeding success) traits were entered as response variables in separate models. Measures of MHC class I and class II diversity were entered as fixed factors or covariates in each model (depending on data structure), while sex was entered as an additional fixed factor and genome‐wide heterozygosity as a covariate. In the analyses of phenotypic traits, we also entered capture date and body size (PC1) as covariates. Hour of measurement was entered as an additional covariate only in the analysis of blood haemoglobin concentration to control for diurnal variation in this trait. In the analyses of clutch size and hatching/breeding success, we entered laying date (covariate) and brood status (fixed factor) to control for variation in reproductive performance between first, renest and second clutches. The analysis of laying dates was conducted for first clutches only. We also tested for the effects of interactions between MHC diversity (class I and class II) and sex on each phenotypic and reproductive trait, to assess whether these associations are sex specific. All interactions were non‐significant and removed from the models, except for the analysis of blood haemoglobin concentration (Tables S1 and S2 in the ESM). Thus, the final models for blood haemoglobin concentration were run separately for each sex. To test for nonlinear patterns in uncategorized MHC allelic diversity, we also added the squared effects of allele numbers (full models), which were subsequently removed if found non‐significant (reduced models). MHC diversity was mean centred in these models (with squared effects). As statistical significance of associations between MHC diversity and reproductive traits may be inflated by pseudoreplication resulting from including both members of a single breeding pair (14.6% of cases), we have rerun the models by randomly subsampling a single birds per pair.

We also tested for the effects of specific MHC supertypes on each fitness‐related trait (not possible for alleles because of their lower frequencies), and these models were run separately for MHC class I and II. Because of numerous predictors (12 MHC class I and 14 MHC class II supertypes, see results for details), we reduced these models by removing highly non‐significant (*p* > 0.15) supertype effects and corrected *p*‐values for multiple comparisons (false discovery rate; Benjamini & Hochberg, 1995). Finally, we tested whether associations of MHC supertype diversity with fitness‐related traits were not primarily driven by the occurrence of specific supertypes and for this purpose we combined both types of predictors (supertype diversity and presence of specific supertypes) within the same models.

The effects of individual identity and year were entered as random factors in each GLMM to avoid pseudoreplication resulting from repeated measurements of the same individuals (e.g. across years) and to control for inter‐annual variation in all the traits. Hatching success (binary trait) was analysed with a binomial distribution, while breeding success was analysed with a zero‐inflated Poisson distribution. All other analyses were run with Gaussian distribution for the response variables. All values are reported as means ± *SE*.

**TABLE 1:** Characteristics of fitness‐related (phenotypic and reproductive) traits in the study population of the Eurasian coot

Trait category	Trait	Sample size (no. measurements/no. individuals)	Mean	*SE*	Range (min – max)
Phenotypic	Blood haemoglobin concentration (g/L)	129/108	150.0	1.6	104–201
	Body mass (g)	129/108	767.7	9.4	559–1017
	Frontal shield width/length (mm)	117/98	20.0/28.7	0.3/0.3	15.2–31.4/23.1–40.6
Reproductive	Laying date (day of year)	191/105	110.8	1.2	80–170
	Clutch size (n eggs)	171/99	7.95	0.13	4–14
	Hatching success	225/103	0.77	0.03	0–1
	Breeding success	204/96	3.50	0.18	0–9

## 3 RESULTS

**FIGURE 1:**
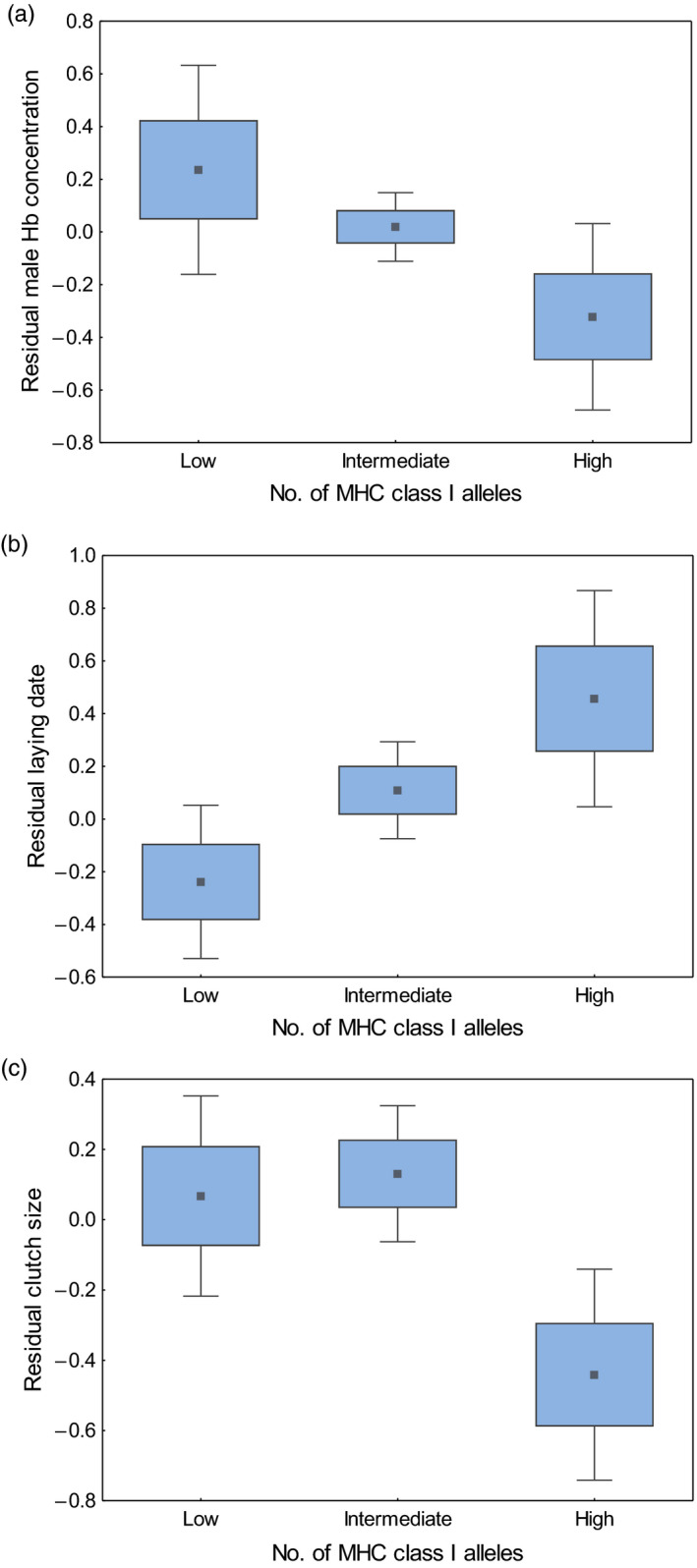
Associations of MHC class I allele numbers with male blood haemoglobin (Hb) concentration (a), laying date (b) and clutch size (c) in the Eurasian coot. Data presented as residuals from GLMMs (Tables 2 and 4). Central point – mean, box – SE and whiskers – 95% confidence intervals

**FIGURE 2:**
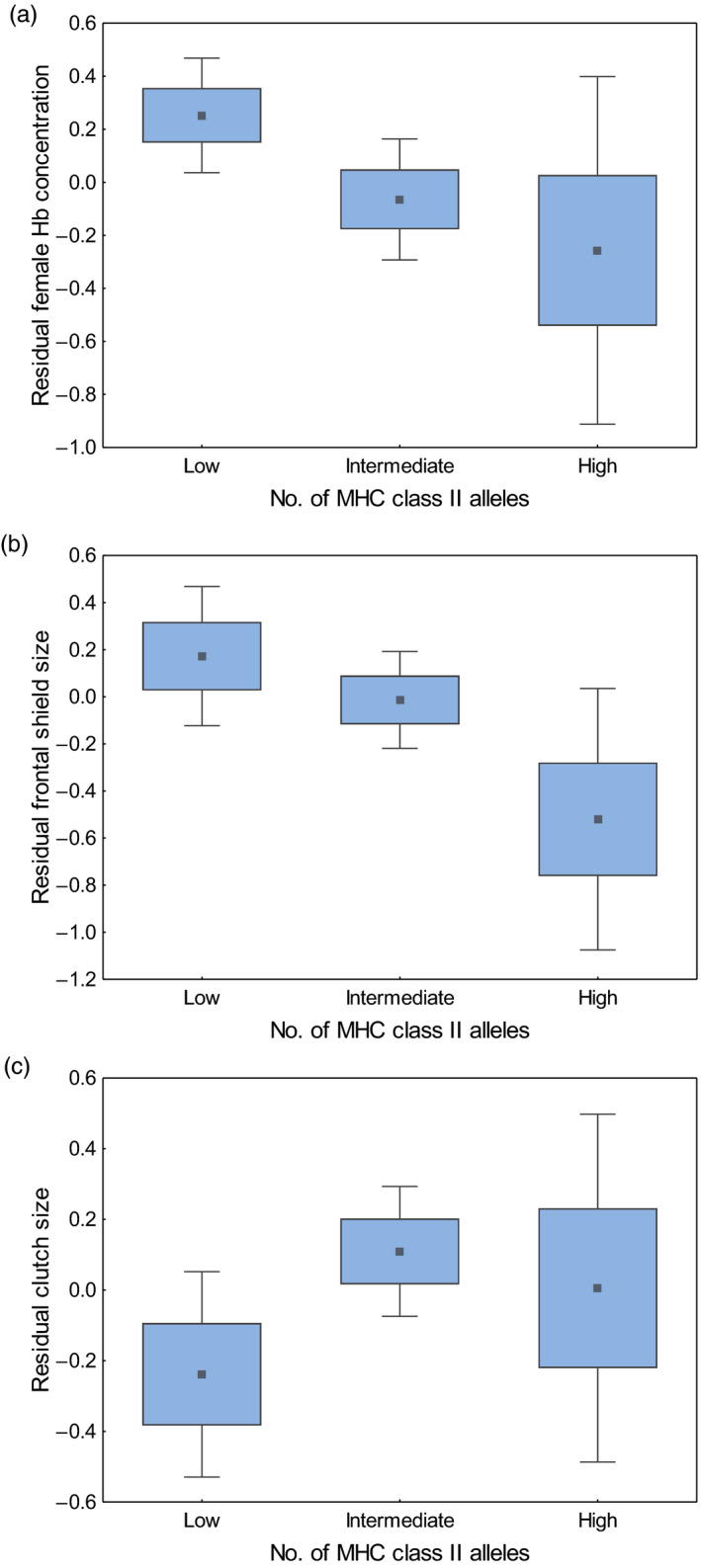
Associations of MHC class II allele numbers with female blood haemoglobin (Hb) concentration (a), frontal shield size (b) and clutch size (c) in the Eurasian coot. Data presented as residuals from GLMMs (Tables 2, 3, 4). Central point – mean, box – SE and whiskers – 95% confidence intervals

The analysis of categorized MHC data revealed significant associations of MHC allelic diversity with physiological condition and ornament expression in the Eurasian coot, but in the opposite direction of our predictions. Birds with a high number of MHC class I and class II alleles were in poorer condition (had significantly lower blood haemoglobin concentrations) when compared with birds that had a low number of MHC alleles, although these associations were sex specific (see Table S1 for significant MHC‐sex interaction) and apparent either in males (MHC class I) or females (MHC class II; Table 2; Figures 1a and 2a). Also, coots with high MHC class II diversity had smaller ornament, the frontal shield (Table 3; Figure 2b). No significant association was found between MHC class I or class II diversity and body mass (after correction for structural size of individuals; Table S2). The analyses of reproductive traits revealed significant relationships between MHC diversity and both laying date and clutch size. Laying date was associated with MHC class I diversity, as individuals with intermediate and high number of MHC alleles started breeding significantly later (on average 5.47 ± 2.59 and 7.34 ± 3.18 days later respectively) than individuals with low number of alleles (Table 4; Figure 1b). Also, coots with high MHC class I diversity had smaller clutches than birds with low MHC diversity, even after controlling for variation in laying date (Table 4, Figure 1c). In contrast, individuals with intermediate MHC class II diversity had significantly larger clutch sizes than individuals with low diversity (Table 4; Figure 2c), whereas no significant differences in clutch size were recorded between birds that had low and high MHC class II diversity (Table 4). Associations of MHC diversity with both laying date and clutch size were apparent in both sexes, as indicated by non‐significant MHC–sex interactions (Table S3). Also, significance of these associations was retained while subsampling a single bird per pair (Table S4). In contrast to laying date and clutch size, we found no evidence for significant associations between MHC diversity and hatching or breeding success (Table S5). Also, we found no statistical support for associations of genome‐wide heterozygosity with either phenotypic traits (condition, ornament expression) or reproductive traits (laying date, clutch size, hatching and breeding success) of coots (Tables 2, 3, 4; Tables S2 and S5).

**TABLE 2:** Associations between haemoglobin concentration and the number of MHC class I and class II alleles in male and female Eurasian coots. Both models included haemoglobin concentration as the response and the number of both class I and II alleles (included separately, each coded with three levels), genome‐wide heterozygosity, body size, capture date and time of sampling (hour) as predictors. Bird identity and year were included as random factors in each model. Reference levels of low MHC class I and II diversity were included in the intercept. Significant predictors are marked in bold

Trait	Predictors	Estimate ± *SE*	*z* value	*p*
Haemoglobin concentration (males)	**Intercept**	**128.66 ± 42.28**	**3.04**	**0.002**
	MHC class I alleles (intermediate vs. low)	−7.37 ± 4.50	1.64	0.10
	**MHC class I alleles (high vs. low)**	**−18.57 ± 4.85**	**3.83**	**<0.001**
	MHC class II alleles (intermediate vs. low)	2.40 ± 4.91	0.49	0.62
	MHC class II alleles (high vs. low)	−11.77 ± 7.73	1.52	0.13
	Genome‐wide heterozygosity	274.74 ± 180.61	1.52	0.13
	Body size	1.12 ± 2.31	0.48	0.63
	**Capture date**	**−0.20 ± 0.05**	**3.92**	**<0.001**
	Hour	0.38 ± 0.86	0.45	0.65
Haemoglobin concentration (females)	**Intercept**	**152.01 ± 44.34**	**3.43**	**0.001**
	MHC class I alleles (intermediate vs. low)	7.23 ± 5.27	1.37	0.17
	MHC class I alleles (high vs. low)	3.70 ± 6.45	0.57	0.57
	**MHC class II alleles (intermediate vs. low)**	**−10.67 ± 5.01**	**2.13**	**0.033**
	**MHC class II alleles (high vs. low)**	**−17.41 ± 7.30**	**2.39**	**0.017**
	Genome‐wide heterozygosity	177.36 ± 208.72	0.85	0.40
	Body size	−5.04 ± 3.26	1.55	0.12
	**Capture date**	**−0.31 ± 0.08**	**3.80**	**<0.001**
	Hour	−0.39 ± 1.01	0.39	0.70

**TABLE 3:** Associations between frontal shield size and the number of MHC class I and class II alleles in the Eurasian coot. The model frontal shield size as the response and the number of both class I and II alleles (included separately, each coded with three levels), genome‐wide heterozygosity, sex, body size and capture date. Bird identity and year were included as random factors in each model. Reference levels of low MHC class I and II diversity were included in the intercept. Significant predictors are marked in bold

Trait	Predictors	Estimate ± *SE*	*z* value	*p*
Frontal shield size	Intercept	3.15 ± 2.00	1.58	0.11
	MHC class I alleles (intermediate vs. low)	−0.16 ± 0.23	0.69	0.49
	MHC class I alleles (high vs. low)	−0.01 ± 0.28	0.02	0.99
	MHC class II alleles (intermediate vs. low)	−0.24 ± 0.23	1.06	0.29
	**MHC class II alleles (high vs. low)**	**−0.86 ± 0.38**	**2.25**	**0.024**
	Genome‐wide heterozygosity	−7.53 ± 9.61	0.78	0.43
	**Sex (male vs. female)**	**1.17 ± 0.35**	**3.34**	**0.001**
	Body size	0.20 ± 0.13	1.51	0.13
	**Capture date**	**−0.014 ± 0.003**	**4.36**	**<0.001**

**TABLE 4:** Associations between two reproductive traits (laying date and clutch size) and the number of MHC class I and class II alleles in the Eurasian coot. The first model included laying date as the response and the number of both class I and II alleles (included separately, each coded with three levels), sex and genome‐wide heterozygosity as predictors. The second model included clutch size as the response and the same predictors with brood status (three levels) and laying date added. Bird identity and year were included as random factors in each model. Reference levels of low MHC class I and II diversity were included in the intercept. Significant predictors are marked in bold

Trait	Predictors	Estimate ± *SE*	*z* value	*p*
Laying date	**Intercept**	**118.29 ± 22.71**	**5.21**	**<0.001**
	**MHC class I alleles (intermediate vs. low)**	**5.51 ± 2.59**	**2.13**	**0.033**
	**MHC class I alleles (high vs. low)**	**7.39 ± 3.18**	**2.33**	**0.020**
	MHC class II alleles (intermediate vs. low)	1.31 ± 2.63	0.50	0.62
	MHC class II alleles (high vs. low)	−3.09 ± 4.57	0.67	0.50
	Genome‐wide heterozygosity	−61.75 ± 114.60	0.54	0.59
	Sex (male vs. female)	0.54 ± 2.39	0.23	0.82
Clutch size	**Intercept**	**13.15 ± 2.51**	**5.23**	**<0.001**
	MHC class I alleles (intermediate vs. low)	0.16 ± 0.25	0.65	0.52
	**MHC class I alleles (high vs. low)**	**−0.88 ± 0.31**	**2.81**	**0.005**
	**MHC class II alleles (intermediate vs. low)**	**0.60 ± 0.26**	**2.34**	**0.019**
	MHC class II alleles (high vs. low)	0.33 ± 0.43	0.76	0.45
	Genome‐wide heterozygosity	−2.46 ± 10.62	0.23	0.82
	Sex (male vs. female)	−0.03 ± 0.23	0.13	0.90
	Brood status (first vs. second)	0.02 ± 1.01	0.02	0.98
	Brood status (renest vs. second)	0.58 ± 1.02	0.57	0.57
	**Laying date**	**−0.04 ± 0.01**	**6.22**	**<0.001**

Consistently, the analysis of uncategorized MHC data provided no support for the benefits of broad MHC repertoire. The MHC class I diversity showed negative linear associations with male haemoglobin concentration (Table S6) and clutch size (Table S7), while a positive linear association was found for laying date (Table S8). The MHC class II diversity showed a significant negative linear association with female haemoglobin concentration (Table S9) and there was a marginally non‐significant (*p* = 0.056) negative association with frontal shield size (Table S10). We also found a negative quadratic relationship between MHC class II diversity and body mass (Table S11), but no associations were recorded between uncategorized MHC allelic diversity and hatching or breeding success (Tables S12 and S13).

Allele clustering indicted the presence of 12 MHC class I and 14 MHC class II supertypes (18.2 ± 4.8 and 10.6 ± 1.1 alleles per supertype respectively) in our study population. After controlling for the false discovery rate, we found that nearly half of all MHC supertypes (46.2%) showed significant associations with different phenotypic or reproductive traits. In particular, we identified five MHC class I supertypes that were associated with reduced body mass and haemoglobin concentration, delayed laying and smaller clutches, while seven MHC class II supertypes were associated with reduced condition (body mass and haemoglobin concentration) and lower breeding success (Tables S14–S27). None of the supertypes were associated with stronger expression of fitness‐related traits (Tables S14–S27). The analysis of MHC supertype data indicated that high supertype diversity was linked with weaker expression of several phenotypic and reproductive traits. Specifically, the number of MHC class I supertypes was negatively associated with male haemoglobin concentration and positively associated with laying date (indicating delayed breeding), while the number of MHC class II supertypes showed negative associations with male body mass and female haemoglobin concentration (Tables S28 and S29). Most of these associations remained significant after controlling for the effects of particular supertypes (Tables S30–S33).

## 4 DISCUSSION

This study examined relationships between the diversity of MHC genes and fitness‐related traits (condition, ornament expression, reproduction) in a recently established urban population of the Eurasian coot. Contrary to expectation, we showed that high allelic diversity of MHC class I or class II genes was associated with poorer condition (lower blood haemoglobin concentrations), weaker expression of the putative ornament (smaller frontal shield), later onset of breeding and smaller clutches. An analysis of functional allele clusters (supertypes) provided further support for negative associations of MHC diversity with phenotypic quality and reproductive performance. We also showed that most of these relationships could not be explained by the presence of specific maladaptive supertypes. Finally, we found little support for the optimality hypothesis, as only body mass and clutch size showed some weak (inconsistent between analyses) evidence of nonlinear associations with MHC class II diversity. Overall, our results indicated that high MHC allelic diversity in our study coot population did not confer any apparent advantage in terms of phenotypic quality or reproductive performance.

So far, a positive relationship between individual MHC diversity and fitness has been empirically supported in several bird species, as high allelic diversity of the MHC was associated with lower pathogen prevalence (Radwan et al., 2012), increased survival (Westerdahl et al., 2005; Worley et al., 2010) or high reproductive success (Brouwer et al., 2010). On the other hand, many bird species show no significant correlation between MHC diversity and infection rate (Loiseau et al., 2008; Sutton et al., 2016) or fitness (Bateson et al., 2016; Ekblom et al., 2004; Radwan et al., 2012). The relative scarcity of positive linear associations may be due to the inherent costs of displaying a wide array of MHC alleles, including an increased risk of autoimmune diseases or depleted repertoire of TCRs that recognize MHC–peptide complexes (Nowak et al., 1992; Todd et al., 1988). In the three‐spined stickleback, individuals with either high or low MHC class II diversity had lower resistance to parasites such as *Schistocephalus solidus* tapeworms and *Glugea anomala* microsporidia, compared to individuals with an intermediate number of MHC alleles (consistent with the optimality hypothesis; Kurtz et al., 2004). This nonlinear pattern may arise from the contradictory mechanisms of heterozygote advantage (a low ability to bind diverse antigens by individuals with low MHC diversity) and TCR repertoire depletion (a low ability to recognize MHC–peptide complexes by TCRs of individuals with high MHC diversity). Our results were not consistent with the optimality hypothesis, as we found little evidence for any reduction in phenotypic quality or reproductive performance of individuals with the lowest MHC diversity. At the same time, we found virtually no support for the diversity hypothesis at the MHC genes in our coot population, as individuals with a high number of MHC alleles or supertypes were characterized by poorer phenotypic quality and reproduction. Finally, our analyses showed that this pattern was not primarily driven by the presence of specific resistance/susceptibility supertypes that could be non‐randomly associated with haplotypes carrying different numbers of MHC variants.

It is possible that the negative associations of MHC diversity with fitness‐related traits could reflect depauperate pathogen diversity in an urban landscape, as urbanization is known to reduce the abundance of many wildlife parasites (Bradley & Altizer, 2007). Under such a scenario, a large number of MHC variants expressed within individuals may not bring the usual benefits in terms of pathogen recognition, while incurring standard costs. Immune defence, in general, is costly in terms of energy or biochemical substrates and while the deployment and maintenance of innate immunity may incur greater costs, development of acquired immunity requires energy and biochemical substrates to fuel the generation of a large pool of T and B lymphocytes with diverse antigen‐binding specificities (McDade et al., 2016). There is also convincing empirical evidence for metabolic costs (e.g. increased basal metabolic rate) of adaptive immunity in birds (Eraud et al., 2005; Ots et al., 2001) and because of trade‐offs in resource allocation, individuals are expected to optimize rather than maximize their immune responses (Sheldon & Verhulst, 1996). Furthermore, an activated immune system extensively produces reactive metabolites and radicals (e.g. through respiratory burst), which are normally directed against pathogens, but may also be detrimental to host tissues (von Schantz et al., 1999). While the MHC expression may be allele‐specific and overall expression levels may not be driven by the levels of allelic diversity per se (Schwensow et al., 2019), individuals with higher MHC diversity are likely to carry a greater number of highly expressed alleles, thus leading to a stronger activation of the immune system. Consequently, reduction of immunological costs (low MHC repertoire) may possibly be adaptive under limited pathogen pressure. In fact, we previously found a marked reduction in MHC class II allele richness (by 34%–48%) within urban coot populations (including the one studied here) when compared to the putative source populations from a non‐urban landscape (Pikus et al., 2021). At the same time, we found no reduction in microsatellite diversity in the urban populations, providing no evidence for genetic bottlenecks and suggesting that limited MHC diversity of urban coots could reflect adaptive processes (Pikus et al., 2021). It remains to be tested whether parasite pressure is reduced in urban coot populations, but the prevalence of different parasite groups (cestodes, trematodes, chewing lice) was reported to be extremely high (80%–100%) in non‐urban coot populations (Yakovleva et al., 2021; Ziani et al., 2020). Many other zoonotic and vector‐borne pathogens (e.g. *Campylobacter* bacteria, Japanese encephalitis virus) also showed high (>60%) prevalences or seroprevalences in coots sampled from non‐urban habitats (Antilles et al., 2015; Yang et al., 2011). While we lacked any empirical data on pathogen and parasite communities of coots from our urban study site and from adjacent wildland, our results clearly show that, under certain conditions, a high diversity of the MHC genes may not provide any apparent advantages in natural populations.

The TCR depletion hypothesis was recently tested in the bank vole *M. glareolus* (Migalska et al., 2019), and it was shown that the higher diversity of MHC class I genes was associated with a reduced TCR repertoire, although a similar relationship was not observed at the MHC class II genes (Migalska et al., 2019). To the best of our knowledge, the mechanism of TCR depletion has not been unequivocally demonstrated in birds so far, but the assumptions of the optimality hypothesis have been tested in several avian species, leading to mixed conclusions. For example, the mechanism of optimal MHC diversity has not been supported in female collared flycatchers *Ficedula albicollis*, as the prevalence of malaria infections showed a negative linear relationship with functional diversity of the MHC class II and there was no evidence for associations between MHC diversity and female survival or reproductive success (Radwan et al., 2012). Research conducted on the black‐legged kittiwake *Rissa tridactyla* chicks showed a positive correlation between the diversity of MHC class II genes and chick growth rate and survival, as well as tick clearance in females but not in males (Pineaux et al., 2020). Despite differences between the sexes that may have arisen from sex‐specific intensity of parasite infections, the results were not consistent with the assumptions of the optimality hypothesis (Pineaux et al., 2020). Similarly, no support for nonlinear relationships between MHC diversity and individual fitness or intensity of malaria infections was found in a population of the great tit *Parus major* (Sepil et al., 2013). In contrast, empirical evidence for the optimality hypothesis in birds is scarce. For example, a Swedish population of the great reed warbler *Acrocephalus arundinaceus* showed a nonlinear (consistent with the optimality hypothesis) relationship between the number of MHC alleles and the occurrence of infection with certain strains of malaria (Westerdahl et al., 2005). Other studies on the great reed warbler seem to partially support the optimality hypothesis, as there was a nonlinear relationship between female reproductive success and the diversity of MHC genes, although no such relationship was found in males (Roved et al., 2018). Taking all this into account, any robust conclusions on the generality of linear and nonlinear relationships between individual MHC diversity and fitness are currently difficult to draw without further studies in a broader phylogenetic context.

In our study, individual MHC diversity correlated with various characteristics that are expected be related to fitness (condition, ornament expression, reproductive phenology and clutch size), but we did not find any direct relationship between the number of MHC alleles and the key fitness component, that is reproductive success. Phenotypic traits such as condition or expression of ornaments can be directly associated with individual immunocompetence (Koch et al., 2017), and therefore they may show relatively strong associations with the MHC. So far, some of the strongest evidence for associations between ornament expression and MHC diversity comes from studies on the common yellowthroat, in which MHC class II diversity correlated positively with the expression of both carotenoid (yellow bib brightness) and melanin‐based (black facial mask size) ornaments (Dunn et al., 2013; Whittingham et al., 2015). Initial phases of the reproductive cycle (e.g. the date of egg laying or the number of eggs laid) should also be more directly dependent on individual health status and condition (and thus indirectly on the abilities of the immune system) than reproductive success, which is more environmentally dependent (e.g. on the intensity of predation pressure or weather conditions; e.g. Descamps et al., 2011; Hasselquist et al., 2001). Hence, the influence of MHC diversity on reproductive success may be much more effectively masked by the impact of external conditions not directly linked to the phenotypic characteristics of individuals or their genetic constitution. In the case of our urban coot population, this impact may be even greater than for populations nesting in natural habitats. Availability of traditional breeding habitat (patches of reed vegetation) is highly limited in urban landscapes, thus the nests are often poorly sheltered and more exposed to urban predators (mainly corvids, but also domestic animals such as dogs) and unfavourable weather conditions. As a result, effect sizes of the association between MHC diversity and reproductive output are expected to be low and possibly difficult to detect under limited sample sizes. So far, the relationship between MHC and bird reproduction has mainly been reported for reproductive traits associated with the early stages of the breeding cycle, for example, egg mass (Hale et al., 2009), clutch size (Bonneaud et al., 2004) or hatching success (Hoover et al., 2018; Knafler et al., 2012), while correlations with fledging success were reported only sporadically (e.g. Agudo et al., 2012).

In conclusion, high individual diversity of the MHC genes is traditionally expected to increase fitness by broadening the spectrum of pathogens recognized by the immune system (e.g. due to the heterozygote advantage mechanism). However, our results suggest that in some populations any advantages of high MHC diversity may be effectively masked by the costs, which can include increased autoimmune reactions, elevated metabolic costs and TCR depletion. The relative balance between these two contrasting mechanisms should produce a scenario in which optimal (intermediate) MHC diversity is associated with the highest fitness. However, our results suggest that the costs of maintaining high MHC diversity may, under certain conditions, outweigh the benefits, resulting in a negative relationship between MHC diversity and phenotypic quality or reproduction. To the best of our knowledge, this is one of the first studies providing consistent evidence for negative associations between MHC diversity and a range of phenotypic and reproductive traits in a natural avian population.

## AUTHORS' CONTRIBUTIONS

E.P. and P.M. designed the study; E.P. and P.M. collected the data; E.P. conducted laboratory analyses; all authors analysed the data; E.P. wrote the manuscript. All authors contributed critically to the drafts and gave final approval for publication.

## FUNDING INFORMATION

The study was financially supported by the research grant of the National Science Centre in Poland (2020/38/E/NZ8/00143).

## CONFLICT OF INTERESTS

The authors declare no conflict of interests.

## Supporting information


Data S1

## Data Availability

Data available from the Dryad Digital Repository https://doi.org/10.5061/dryad.ttdz08m1j (Pikus et al., 2022). All novel MHC sequences have been deposited in GenBank (accession nos. ON075844‐ON076008).
