# Supplementary material for: High MHC diversity confers no advantage for phenotypic quality and reproductive performance in a wild bird
Source: J Anim Ecol. 2022 May 15;91(8):1707–18. doi: 10.1111/1365-2656.13737 (PMC9542035; doi:10.1111/1365-2656.13737)

Electronic Supplementary Material

High MHC diversity confers no advantage for phenotypic quality and reproductive performance in a wild bird

Ewa Pikus[1], Peter O. Dunn[2], Piotr Minias[1]

[1] Department of Biodiversity Studies and Bioeducation, Faculty of Biology and Environmental Protection, University of Łódź, Banacha 1/3, 90-237 Łódź, Poland

[2] Behavioral and Molecular Ecology Group, Department of Biological Sciences, University of Wisconsin-Milwaukee

Author for correspondence

Piotr Minias

Email: pminias@op.pl

Supplementary methods

Processing of Illumina data and MHC allele validation

First, paired-end sequences were merged using the Amplicon Sequencing MERGing tool (AmpliMERGE), which operates based on the FLASH algorithm with optimum overlapping parameters (Magoč & Salzberg, 2011). The stages of de-multiplexing, clustering, and filtering of merged reads were conducted in the Amplicon Sequencing Assignment (AmpliSAS) tool. At the clustering stage we used default parameters for Illumina data, including 1% substitution errors, 0.001% indel errors, and 25% minimum dominant frequency. During the filtering stage, chimeras and low frequency sequences (>3%) were discarded, as recommended for the general processing of Illumina data (Kircher et al., 2011). The processing was conducted for amplicons with the minimum depth of 300 reads and the maximum amplicon depth was set to 5000 reads because of AmpliSAS performance reasons (by default excluding additional reads from analyses). The average amplicon depth was 1706 ± 73 [SE] (prior to processing) and the average number of reads for validated sequences was 1398 ± 64 [SE] reads per sample. To determine the reproducibility of our genotyping and processing approaches, a total of 36 technical replicates (samples from the same individuals amplified in independent PCR reactions) were genotyped. The technical reproducibility (concordance rate) of validated sequences was high (89.3% and 95.4% for MHC class I and II, respectively), as calculated following Qi et al. (2015).

Supertype clustering

Supertype inference was performed separately for MHC class I and class II alleles using k-means clustering and discriminant function analysis of principal components (DAPC) implemented in *adegenet* R package (Jombart, 2008), following methodology by Biedrzycka et al. (2018). The analysis was limited to the positively selected sites (as identified for the Eurasian coot by Pikus & Minias 2022), which are likely corresponding to the peptide-binding region of the MHC molecules. Since the number of sites under pervasive positive selection was much lower at class I (n = 8) than class II (n = 18), we used sites under both episodic and pervasive selection for MHC class I (n = 15) (Pikus & Minias, 2022). To infer supertypes, we first identified the most probable number of clusters based on Bayesian information criterion (BIC), choosing the minimal number of clusters that preceded any increase in BIC values. This approach indicated the presence of 12 clusters for MHC class I and 14 clusters for MHC class II (Fig. S1). The number of principal components (PCs) in DAPC analysis was selected based on the maximum α-score (identified with the *optim.a.score* function), resulting in

seven PCs in each analysis (Fig. S2). We used all available discriminant functions (n = 7) and re-ran the entire procedure ten times to assess repeatability of supertype clustering. Repeatability was high for both MHC class I (R = 0.813, P < 0.001) and MHC class II (R = 0.827, P < 0.001), as assessed with kappa value (Fleiss, 1971) implemented in the *irr* R package (Gamer et al., 2012). The mean assignment probabilities (across all runs) were high for MHC class I (0.992 ± 0.001 [SE]) and MHC class II (0.983 ± 0.002 [SE]).

ddRAD sequencing

In the ddRAD sequencing we used PstI (5'-CTGCA/G-3') and MboI (5'-/GATC-3') restriction enzymes for DNA digestion and selected 300-600 bp fragments for Illumina HiSeq 2500v4 sequencing. Approximately 2.3 million reads per sample were generated and processed with Stacks 2.0 software (Catchen et al., 2013). The processing included the following steps: *i*) trimming and demultiplexing raw reads; *ii*) removing low quality reads (based onPhred33 quality score) and reads with uncalled sites; *iii*) calling single nucleotide polymorphism (SNP) with de novo approach (a minimum coverage depth of 3 reads per stack and a maximum of 3 mismatches between any two alleles in the population and within individuals). The processing stage produced 868 297 loci with an effective sample coverage of 9x (SD = 1.8x, range: 5.0 – 14.7x), which were then exported into a variant call format (VCF) file. Genome-wide heterozygosity was calculated across 14 525 SNPs that were called in at least 80% of individuals (on average 12 831 ± 98 [SE] SNPs per sample) using VCFtools (Danecek et al., 2011).

References

Biedrzycka, A., Bielański, W., Ćmiel, A., Solarz, W., Zając, T., Migalska, M., Sebastian, A., Westerdahl, H., Radwan, J. (2018). Blood parasites shape extreme major histocompatibility complex diversity in a migratory passerine. *Molecular Ecology*, 27(11), 2594-2603.

Catchen, J., Hohenlohe, P. A., Bassham, S., Amores, A., & Cresko, W. A. (2013). Stacks: an analysis tool set for population genomics. *Molecular Ecology*, *22*(11), 3124–3140.

Danecek, P., Auton, A., Abecasis, G., Albers, C. A., Banks, E., DePristo, M. A., Handsaker, R. E., Lunter, G., Marth, G. T., Sherry, S. T., McVean, G., & Durbin, R. (2011). The variant call format and VCFtools. *Bioinformatics*, *27*(15), 2156–2158.

Fleiss, J. L. (1971). Measuring nominal scale agreement among many raters. *Psychological Bulletin*, *76*(5), 378-382.

Gamer, M., Lemon, J., Fellows, I., & Singh, P. (2012). irr: Various coefficients for interrater reliability and agreement. R package ver. 0.84. Available at: https://CRAN.R-project.org/package=irr

Jombart, T. (2008). adegenet: a R package for the multivariate analysis of genetic markers. *Bioinformatics*, *24*(11), 1403-1405.

Kircher, M., Heyn, P., & Kelso, J. (2011). Addressing challenges in the production and analysis of Illumina sequencing data. *BMC Genomics*, *12*(1), 382.

Magoč, T., & Salzberg, S. L. (2011). FLASH: fast length adjustment of short reads to improve genome assemblies. *Bioinformatics*, *27*(21), 2957-2963.

Pikus, E., Minias, P. (2022) Using de novo genome assembly and high-throughput sequencing to characterize the Major Histocompatibility Complex in a non-model rallid bird, the Eurasian coot *Fulica atra*. *Scientific Reports*, *12*, 7031.

Qi, Y., Liu, X., Liu, C. G., Wang, B., Hess, K. R., Symmans, W. F., Shi, W., & Pusztai, L. (2015). Reproducibility of variant calls in replicate next generation sequencing experiments. *PLoS ONE*, *10*(7), e0119230.

Table S1. Models assessing the effects of interactions between the number of MHC class I and class II alleles (categorized data) and sex on three phenotypic traits (body mass, haemoglobin concentration and frontal shield size) in the Eurasian coot. Bird identity and year were included as random factors in each model. Wald χ^2 statistics were used to assess statistical significance and significant predictors are marked in bold.

Trait	Predictors	W	P
Body mass	**Intercept**	**295.12**	**<0.001**
	MHC class I alleles	2.80	0.25
	MHC class II alleles	0.79	0.67
	Genome-wide heterozygosity	3.33	0.068
	**Sex**	**9.13**	**0.003**
	**Body size**	**18.90**	**<0.001**
	Capture date	2.93	0.087
	MHC class I alleles * Sex	3.78	0.15
	MHC class II alleles * Sex	0.96	0.62
Haemoglobin concentration	Intercept	**24.37**	**<0.001**
	MHC class I alleles	2.94	0.23
	**MHC class II alleles**	**9.29**	**0.010**
	Genome-wide heterozygosity	1.73	0.19
	**Sex**	**5.61**	**0.018**
	Body size	0.84	0.36
	**Capture date**	**32.73**	**<0.001**
	Hour	0.10	0.76
	**MHC class I alleles * Sex**	**12.92**	**0.002**
	MHC class II alleles * Sex	2.74	0.25
Frontal shield size	Intercept	2.71	0.10
	MHC class I alleles	1.52	0.47
	MHC class II alleles	4.96	0.084
	Genome-wide heterozygosity	0.54	0.46
	Sex	3.73	0.053
	Body size	1.55	0.21
	**Capture date**	**22.80**	**<0.001**
	MHC class I alleles * Sex	2.35	0.31
	MHC class II alleles * Sex	2.25	0.33

Table S2. Associations between body mass and the number of MHC class I and class II alleles (categorized data) in the Eurasian coot. The model included body mass as the response and the number of both class I and II alleles (included separately, each coded with three levels), genome-wide heterozygosity, sex, body size, and capture date as predictors. Bird identity and year were included as random factors. Reference levels of low MHC class I and II diversity were included in the intercept. Significant predictors are marked in bold.

Predictors	Estimate ± SE	z value	P
**Intercept**	**30.47 ± 1.83**	**16.64**	**<0.001**
MHC class I alleles (intermediate vs. low)	-0.04 ± 0.21	0.18	0.86
MHC class I alleles (high vs. low)	-0.07 ± 0.26	0.28	0.78
MHC class II alleles (intermediate vs. low)	-0.14 ± 0.22	0.63	0.53
MHC class II alleles (high vs. low)	-0.10 ± 0.33	0.32	0.75
Genome-wide heterozygosity	-14.54 ± 8.91	1.63	0.10
**Sex (male vs. female)**	**2.03 ± 0.33**	**6.07**	**<0.001**
**Body size**	**0.55 ± 0.13**	**4.22**	**<0.001**
Capture date	-0.006 ± 0.003	1.76	0.078

Table S3. Models assessing the effects of interactions between the number of MHC class I and class II alleles (categorized data) and sex on four reproductive traits (laying date, clutch size, hatching and breeding success) in the Eurasian coot. Bird identity and year were included as random factors in each model. Wald χ^2 statistics were used to assess statistical significance and significant predictors are marked in bold.

Trait	Predictors	W	P
Laying date	**Intercept**	**23.27**	**<0.001**
	MHC class I alleles	1.94	0.38
	MHC class II alleles	3.46	0.18
	Genome-wide heterozygosity	0.21	0.64
	Sex	0.37	0.54
	MHC class I alleles * Sex	0.93	0.63
	MHC class II alleles * Sex	3.11	0.21
Clutch size	**Intercept**	**25.79**	**<0.001**
	MHC class I alleles	3.96	0.14
	MHC class II alleles	1.29	0.52
	Genome-wide heterozygosity	0.07	0.79
	Sex	0.01	0.91
	Brood status	2.31	0.31
	**Laying date**	**35.67**	**<0.001**
	MHC class I alleles * Sex	0.36	0.83
	MHC class II alleles * Sex	0.45	0.80
Hatching success	Intercept	1.02	0.31
	MHC class I alleles	0.09	0.96
	MHC class II alleles	0.75	0.69
	Genome-wide heterozygosity	0.63	0.43
	Sex	0.50	0.48
	Brood status	1.69	0.43
	Laying date	0.36	0.55
	MHC class I alleles * Sex	0.64	0.72
	MHC class II alleles * Sex	0.09	0.96
Breeding success	Intercept	0.35	0.55
	MHC class I alleles	2.06	0.36
	MHC class II alleles	0.03	0.99
	Genome-wide heterozygosity	2.49	0.11
	Sex	1.02	0.31
	Brood status	0.98	0.61
	**Laying date**	**7.72**	**0.005**
	MHC class I alleles * Sex	0.90	0.64
	MHC class II alleles * Sex	0.56	0.76

Table S4. Associations between two reproductive traits (laying date and clutch size) and the number of MHC class I and class II alleles in the Eurasian coot after random subsampling of a single bird per pair (n = 163 individuals for laying date and n = 139 individuals for clutch size). The first model included laying date as the response and the number of both class I and II alleles (included separately, each coded with three levels), sex and genome-wide heterozygosity as predictors. The second model included clutch size as the response and the same predictors with brood status (three levels) and laying date added. Bird identity and year were included as random factors in each model. Reference levels of low MHC class I and II diversity were included in the intercept. Significant predictors are marked in bold.

Trait	Predictors	Estimate ± SE	z value	P
Laying date	**Intercept**	**129.71 ± 25.23**	**5.14**	**<0.001**
	**MHC class I alleles (intermediate vs. low)**	**5.80 ± 2.79**	**2.08**	**0.038**
	**MHC class I alleles (high vs. low)**	**8.20 ± 3.64**	**2.25**	**0.024**
	MHC class II alleles (intermediate vs. low)	1.59 ± 2.84	0.56	0.58
	MHC class II alleles (high vs. low)	-2.32 ± 5.12	0.45	0.65
	Genome-wide heterozygosity	-122.2 ± 127.5	0.96	0.34
	Sex (male vs. female)	1.49 ± 2.73	0.55	0.59
Clutch size	**Intercept**	**12.51 ± 2.64**	**4.75**	**<0.001**
	MHC class I alleles (intermediate vs. low)	0.30 ± 0.27	1.11	0.27
	**MHC class I alleles (high vs. low)**	**-0.90 ± 0.35**	**2.55**	**0.011**
	**MHC class II alleles (intermediate vs. low)**	**0.54 ± 0.27**	**1.96**	**0.050**
	MHC class II alleles (high vs. low)	0.22 ± 0.50	0.44	0.66
	Genome-wide heterozygosity	-0.86 ± 11.25	0.08	0.94
	Sex (male vs. female)	-0.002 ± 0.250	0.01	0.99
	Brood status (first vs. second)	-0.25 ± 1.01	0.24	0.81
	Brood status (renest vs. second)	-0.38 ± 1.03	0.37	0.71
	**Laying date**	**-0.04 ± 0.01**	**5.29**	**<0.001**

Table S5. Associations between two reproductive traits (hatching and breeding success) and the number of MHC class I and class II alleles (categorized data) in the Eurasian coot. The first model included hatching success as the response and the number of both class I and II alleles (included separately, each coded with three levels), genome-wide heterozygosity, sex, brood status (three levels), and laying date as predictors. The second model included breeding success as the response and the same predictors. Bird identity and year were included as random factors in each model. Reference levels of low MHC class I and II diversity were included in the intercept. Significant predictors are marked in bold.

Trait	Predictors	Estimate ± SE	z value	P
Hatching success	Intercept	4.61 ± 4.17	1.11	0.27
	MHC class I alleles (intermediate vs. low)	-0.19 ± 0.39	0.47	0.64
	MHC class I alleles (high vs. low)	-0.14 ± 0.46	0.29	0.77
	MHC class II alleles (intermediate vs. low)	-0.52 ± 0.41	1.26	0.21
	MHC class II alleles (high vs. low)	0.62 ± 0.84	0.74	0.46
	Genome-wide heterozygosity	-15.69 ± 18.36	0.85	0.39
	Sex (male vs. female)	0.16 ± 0.36	0.44	0.66
	Brood status (first vs. second)	-0.67 ± 1.19	0.57	0.57
	Brood status (renest vs. second)	0.08 ± 1.22	0.06	0.95
	Laying date	0.006 ± 0.010	0.61	0.55
Breeding success	Intercept	0.93 ± 1.20	0.77	0.44
	MHC class I alleles (intermediate vs. low)	0.03 ± 0.12	0.28	0.78
	MHC class I alleles (high vs. low)	0.22 ± 0.14	1.55	0.12
	MHC class II alleles (intermediate vs. low)	-0.10 ± 0.13	0.82	0.41
	MHC class II alleles (high vs. low)	-0.06 ± 0.18	0.35	0.73
	Genome-wide heterozygosity	7.37 ± 4.98	1.48	0.14
	Sex (male vs. female)	0.05 ± 0.11	0.46	0.65
	Brood status (first vs. second)	0.11 ± 0.34	0.32	0.75
	Brood status (renest vs. second)	0.28 ± 0.32	0.88	0.38
	**Laying date**	**-0.011 ± 0.004**	**2.73**	**0.006**

Table S6. Associations between male haemoglobin concentration and the number of MHC class I and class II alleles (mean centred uncategorized data) in the Eurasian coot. The full model (effects of squared MHC diversity included) and reduced model (non-significant effects of squared MHC diversity removed) are presented. Bird identity and year were included as random factors in each model. Significant predictors are marked in bold.

Predictors	Estimate ± SE	z value	P
Full model			
**Intercept**	**126.85 ± 45.92**	**2.76**	**0.006**
**MHC class I alleles**	**-3.00 ± 0.93**	**3.22**	**0.001**
MHC class I alleles squared	0.11 ± 0.30	0.38	0.70
MHC class II alleles	-2.54 ± 2.97	0.86	0.39
MHC class II alleles squared	-1.19 ± 1.60	0.74	0.46
Genome-wide heterozygosity	277.41 ± 192.67	1.44	0.15
Body size	0.25 ± 2.43	0.10	0.91
**Capture date**	**-0.21 ± 0.05**	**3.98**	**<0.001**
Hour	0.07 ± 0.96	0.07	0.94
Reduced model			
**Intercept**	**141.11 ± 42.80**	**3.30**	**<0.001**
**MHC class I alleles**	**-2.71 ± 0.74**	**3.66**	**<0.001**
MHC class II alleles	-2.92 ± 2.66	1.10	0.27
Genome-wide heterozygosity	230.48 ± 183.49	1.26	0.21
Body size	0.05 ± 2.40	0.02	0.98
**Capture date**	**-0.22 ± 0.05**	**4.19**	**<0.001**
Hour	-0.21 ± 0.88	0.24	0.81

Table S7. Associations between clutch size and the number of MHC class I and class II alleles (mean centred uncategorized data) in the Eurasian coot. The full model (effects of squared MHC diversity included) and reduced model (non-significant effects of squared MHC diversity removed) are presented. Bird identity and year were included as random factors in each model. Significant predictors are marked in bold.

Predictors	Estimate ± SE	z value	P
Full model			
**Intercept**	**13.64 ± 2.76**	**4.94**	**<0.001**
MHC class I alleles	-0.07 ± 0.06	1.32	0.19
MHC class I alleles squared	-0.015 ± 0.018	0.82	0.41
MHC class II alleles	0.22 ± 0.16	1.39	0.16
MHC class II alleles squared	-0.04 ± 0.11	0.34	0.73
Genome-wide heterozygosity	-3.29 ± 11.42	0.29	0.77
Sex (male vs. female)	0.04 ± 0.24	0.19	0.85
Brood status (first vs. second)	-0.06 ± 1.04	0.06	0.96
Brood status (renest vs. second)	0.56 ± 1.05	0.53	0.60
**Laying date**	**-0.042 ± 0.007**	**5.94**	**<0.001**
Reduced model			
**Intercept**	**12.99 ± 2.65**	**4.90**	**<0.001**
**MHC class I alleles**	**-0.10 ± 0.05**	**2.05**	**0.040**
MHC class II alleles	0.20 ± 0.16	1.25	0.21
Genome-wide heterozygosity	-1.00 ± 11.12	0.09	0.93
Sex (male vs. female)	0.02 ± 0.24	0.07	0.94
Brood status (first vs. second)	0.02 ± 1.03	0.02	0.98
Brood status (renest vs. second)	0.65 ± 1.04	0.62	0.53
**Laying date**	**-0.042 ± 0.007**	**5.91**	**<0.001**

Table S8. Associations between laying date and the number of MHC class I and class II alleles (mean centred uncategorized data) in the Eurasian coot. The full model (effects of squared MHC diversity included) and reduced model (non-significant effects of squared MHC diversity removed) are presented. Bird identity and year were included as random factors in each model. Significant predictors are marked in bold.

Predictors	Estimate ± SE	z value	P
Full model			
**Intercept**	**122.66 ± 24.03**	**5.11**	**<0.001**
**MHC class I alleles**	**1.41 ± 0.60**	**2.36**	**0.018**
MHC class I alleles squared	-0.12 ± 0.19	0.65	0.51
MHC class II alleles	0.48 ± 1.65	0.29	0.77
MHC class II alleles squared	0.23 ± 1.12	0.21	0.84
Genome-wide heterozygosity	-58.46 ± 117.99	0.50	0.62
Sex (male vs. female)	0.69 ± 2.38	0.29	0.77
Reduced model			
**Intercept**	**118.52 ± 23.04**	**5.14**	**<0.001**
**MHC class I alleles**	**1.18 ± 0.49**	**2.43**	**0.015**
MHC class II alleles	0.30 ± 1.63	0.19	0.85
Genome-wide heterozygosity	-39.55 ± 114.34	0.35	0.73
Sex (male vs. female)	0.49 ± 2.37	0.21	0.84

Table S9. Associations between female haemoglobin concentration and the number of MHC class I and class II alleles (mean centred uncategorized data) in the Eurasian coot. The full model (effects of squared MHC diversity included) and reduced model (non-significant effects of squared MHC diversity removed) are presented. Bird identity and year were included as random factors in each model. Significant predictors are marked in bold.

Predictors	Estimate ± SE	z value	P
Full model			
**Intercept**	**146.53 ± 45.74**	**3.20**	**<0.001**
MHC class I alleles	0.80 ± 1.20	0.66	0.51
MHC class I alleles squared	-0.29 ± 0.37	0.79	0.43
**MHC class II alleles**	**-9.38 ± 3.04**	**3.09**	**0.002**
MHC class II alleles squared	1.05 ± 2.66	0.39	0.69
Genome-wide heterozygosity	202.03 ± 211.07	0.96	0.34
Body size	-4.62 ± 3.25	1.42	0.15
**Capture date**	**-0.34 ± 0.08**	**4.29**	**<0.001**
Hour	-0.22 ± 0.98	0.22	0.82
Reduced model			
**Intercept**	**141.24 ± 44.70**	**3.16**	**<0.001**
MHC class I alleles	0.21 ± 1.00	0.21	0.83
**MHC class II alleles**	**-9.24 ± 3.03**	**3.05**	**0.002**
Genome-wide heterozygosity	227.77 ± 208.81	1.09	0.28
Body size	-4.61 ± 3.20	1.44	0.15
**Capture date**	**-0.34 ± 0.08**	**4.40**	**<0.001**
Hour	-0.18 ± 0.97	0.19	0.85

Table S10. Associations between frontal shield size and the number of MHC class I and class II alleles (mean centred uncategorized data) in the Eurasian coot. The full model (effects of squared MHC diversity included) and reduced model (non-significant effects of squared MHC diversity removed) are presented. Bird identity and year were included as random factors in each model. Significant predictors are marked in bold.

Predictors	Estimate ± SE	z value	P
Full model			
Intercept	2.00 ± 2.09	0.96	0.34
MHC class I alleles	-0.052 ± 0.052	1.00	0.32
MHC class I alleles squared	0.021 ± 0.016	1.31	0.19
MHC class II alleles	-0.28 ± 0.57	1.85	0.064
MHC class II alleles squared	-0.09 ± 0.09	0.96	0.34
Genome-wide heterozygosity	-4.36 ± 9.89	0.44	0.66
**Sex (male vs. female)**	**1.21 ± 0.35**	**3.49**	**<0.001**
Body size	0.20 ± 0.13	1.47	0.14
**Capture date**	**-0.013 ± 0.003**	**4.05**	**<0.001**
Reduced model			
Intercept	2.80 ± 2.03	1.38	0.17
MHC class I alleles	-0.004 ± 0.042	0.09	0.93
MHC class II alleles	-0.28 ± 0.15	1.91	0.056
Genome-wide heterozygosity	-7.54 ± 9.77	0.77	0.44
**Sex (male vs. female)**	**1.24 ± 0.35**	**3.54**	**<0.001**
Body size	0.19 ± 0.14	1.42	0.16
**Capture date**	**-0.014 ± 0.003**	**4.30**	**<0.001**

Table S11. Associations between body mass and the number of MHC class I and class II alleles (mean centred uncategorized data) in the Eurasian coot. The full model (effects of squared MHC diversity included) and reduced model (non-significant effects of squared MHC diversity removed) are presented. Bird identity and year were included as random factors in each model. Significant predictors are marked in bold.

Predictors	Estimate ± SE	z value	P
Full model			
**Intercept**	**30.97 ± 1.83**	**16.95**	**<0.001**
MHC class I alleles	-0.037 ± 0.046	0.81	0.42
MHC class I alleles squared	-0.015 ± 0.014	1.07	0.29
MHC class II alleles	-0.044 ± 0.132	0.33	0.74
**MHC class II alleles squared**	**-0.250 ± 0.082**	**3.04**	**0.002**
Genome-wide heterozygosity	-16.57 ± 8.74	1.90	0.058
**Sex (male vs. female)**	**2.05 ± 0.32**	**6.47**	**<0.001**
**Body size**	**0.56 ± 0.13**	**4.49**	**<0.001**
Capture date	-0.006 ± 0.003	1.90	0.058
Reduced model			
Intercept	30.37 ± 1.74	17.40	<0.001
MHC class I alleles	-0.064 ± 0.038	1.67	0.10
MHC class II alleles	-0.074 ± 0.129	0.57	0.57
**MHC class II alleles squared**	**-0.247 ± 0.082**	**3.00**	**0.003**
Genome-wide heterozygosity	-14.25 ± 8.50	1.68	0.09
**Sex (male vs. female)**	**2.03 ± 0.32**	**6.39**	**<0.001**
**Body mass**	**0.56 ± 0.13**	**4.48**	**<0.001**
Capture date	-0.006 ± 0.003	1.78	0.07

Table S12. Associations between hatching success and the number of MHC class I and class II alleles (mean centred uncategorized data) in the Eurasian coot. The full model (effects of squared MHC diversity included) and reduced model (non-significant effects of squared MHC diversity removed) are presented. Bird identity and year were included as random factors in each model.

Predictors	Estimate ± SE	z value	P
Full model			
Intercept	5.05 ± 4.45	1.14	0.26
MHC class I alleles	-0.002 ± 0.088	0.02	0.98
MHC class I alleles squared	-0.008 ± 0.028	0.27	0.79
MHC class II alleles	0.05 ± 0.29	0.17	0.87
MHC class II alleles squared	0.26 ± 0.27	0.95	0.34
Genome-wide heterozygosity	-18.99 ± 19.07	1.00	0.32
Sex (male vs. female)	0.10 ± 0.36	0.28	0.78
Brood status (first vs. second)	-0.75 ± 1.20	0.63	0.53
Brood status (renest vs. second)	-0.04 ± 1.22	0.03	0.97
Laying date	0.005 ± 0.011	0.45	0.65
Reduced model			
Intercept	4.89 ± 4.29	1.14	0.25
MHC class I alleles	-0.029 ± 0.072	0.40	0.69
MHC class II alleles	0.01 ± 0.25	0.05	0.96
Genome-wide heterozygosity	-17.72 ± 18.41	0.96	0.34
Sex (male vs. female)	0.03 ± 0.35	0.09	0.93
Brood status (first vs. second)	-0.74 ± 1.20	0.61	0.54
Brood status (renest vs. second)	-0.06 ± 1.22	0.05	0.96
Laying date	0.005 ± 0.011	0.44	0.66

Table S13. Associations between breeding success and the number of MHC class I and class II alleles (mean centred uncategorized data) in the Eurasian coot. The full model (effects of squared MHC diversity included) and reduced model (non-significant effects of squared MHC diversity removed) are presented. Bird identity and year were included as random factors in each model. Significant predictors are marked in bold.

Predictors	Estimate ± SE	z value	P
Full model			
Intercept	0.76 ± 1.20	0.63	0.53
MHC class I alleles	0.028 ± 0.026	1.10	0.27
MHC class I alleles squared	-0.007 ± 0.008	0.80	0.42
MHC class II alleles	0.025 ± 0.079	0.31	0.75
MHC class II alleles squared	0.021 ± 0.046	0.47	0.64
Genome-wide heterozygosity	6.01 ± 5.06	1.19	0.24
Sex (male vs. female)	0.01 ± 0.10	0.05	0.96
Brood status (first vs. second)	0.27 ± 0.32	0.83	0.41
Brood status (renest vs. second)	0.26 ± 0.31	0.83	0.41
**Laying date**	**-0.008 ± 0.004**	**2.15**	**0.032**
Reduced model			
Intercept	0.81 ± 1.23	0.66	0.51
MHC class I alleles	0.022 ± 0.022	1.04	0.30
MHC class II alleles	-0.026 ± 0.075	0.35	0.73
Genome-wide heterozygosity	7.32 ± 5.06	1.45	0.15
Sex (male vs. female)	0.03 ± 0.11	0.30	0.77
Brood status (first vs. second)	0.13 ± 0.34	0.39	0.70
Brood status (renest vs. second)	0.27 ± 0.32	0.83	0.41
**Laying date**	**-0.010 ± 0.004**	**2.53**	**0.011**

Table S14. Associations between body mass and presence of specific MHC class I supertypes in the Eurasian coot. Bird identity and year were included as random factors in each model. The results of full and reduced model are shown. Significant predictors are marked in bold (at P < 0.05) or with asterisks (after correction for the false discovery rate).

Predictors	Estimate ± SE	z value	P
Full model			
**Intercept**	**31.47 ± 1.93**	**16.27**	**<0.001***
Supertype MHC1_1	0.15 ± 0.22	0.67	0.51
Supertype MHC1_2	0.12 ± 0.28	0.44	0.66
**Supertype MHC1_3**	**-0.79 ± 0.26**	**-3.03**	**<0.001***
Supertype MHC1_4	0.27 ± 0.24	1.13	0.26
Supertype MHC1_5	0.28 ± 0.32	0.86	0.39
Supertype MHC1_6	0.06 ± 0.33	0.18	0.86
Supertype MHC1_7	-0.39 ± 0.27	-1.47	0.14
Supertype MHC1_8	-0.26 ± 0.25	-1.04	0.30
Supertype MHC1_9	0.18 ± 0.19	0.95	0.34
Supertype MHC1_10	0.22 ± 0.29	0.76	0.45
Supertype MHC1_11	-0.02 ± 0.34	-0.05	0.96
Supertype MHC1_12	0.10 ± 0.28	0.37	0.71
**Genome-wide heterozygosity**	**-19.35 ± 9.29**	**-2.08**	**0.037**
**Sex (male vs. female)**	**2.07 ± 0.36**	**5.82**	**<0.001***
**Body size**	**0.50 ± 0.14**	**3.66**	**<0.001***
**Capture date**	**-0.008 ± 0.003**	**-2.39**	**0.020**
Reduced model			
**Intercept**	**31.51 ± 1.83**	**17.25**	**<0.001***
**Supertype MHC1_3**	**-0.66 ± 0.25**	**-2.67**	**0.008***
Supertype MHC1_7	-0.20 ± 0.24	-0.82	0.41
**Genome-wide heterozygosity**	**-18.95 ± 8.76**	**-2.16**	**0.031***
**Sex (male vs. female)**	**1.99 ± 0.33**	**6.04**	**<0.001***
**Body size**	**0.53 ± 0.13**	**4.16**	**<0.001***
**Capture date**	**-0.007 ± 0.003**	**-2.06**	**0.039***

Table S15. Associations between body mass and presence of specific MHC class II supertypes in the Eurasian coot. Bird identity and year were included as random factors in each model. The results of full and reduced model are shown. Significant predictors are marked in bold (at P < 0.05) or with asterisks (after correction for the false discovery rate).

Predictors	Estimate ± SE	z value	P
Full model			
**Intercept**	**28.82 ± 1.79**	**16.13**	**<0.001***
**Supertype MHC2_1**	**-0.6 ± 0.27**	**-2.25**	**0.024**
Supertype MHC2_2	-0.33 ± 0.26	-1.27	0.20
**Supertype MHC2_3**	**-0.94 ± 0.39**	**-2.43**	**0.015**
Supertype MHC2_4	-0.24 ± 0.31	-0.77	0.44
Supertype MHC2_5	0.15 ± 0.52	0.29	0.77
Supertype MHC2_6	0.14 ± 0.26	0.54	0.59
Supertype MHC2_7	-0.39 ± 0.23	-1.70	0.088
Supertype MHC2_8	-0.59 ± 0.33	-1.78	0.075
Supertype MHC2_9	0.25 ± 0.31	0.83	0.41
Supertype MHC2_10	0.40 ± 0.26	1.52	0.13
Supertype MHC2_11	0.02 ± 0.22	0.07	0.94
Supertype MHC2_12	-0.25 ± 0.30	-0.82	0.41
Supertype MHC2_13	0.19 ± 0.30	0.63	0.53
Supertype MHC2_14	0.09 ± 0.21	0.42	0.67
Genome-wide heterozygosity	-4.85 ± 8.79	-0.55	0.58
**Sex (male vs. female)**	**1.80 ± 0.31**	**5.81**	**<0.001***
**Body size**	**0.52 ± 0.12**	**4.24**	**<0.001***
Capture date	-0.005 ± 0.003	-1.71	0.086
Reduced model			
**Intercept**	**28.96 ± 1.80**	**16.09**	**<0.001***
**Supertype MHC2_1**	**-0.66 ± 0.19**	**-3.44**	**0.001***
**Supertype MHC2_3**	**-0.93 ± 0.37**	**-2.51**	**0.012***
**Supertype MHC2_7**	**-0.40 ± 0.19**	**-2.12**	**0.034**
**Supertype MHC2_8**	**-0.56 ± 0.28**	**-2.02**	**0.043**
Supertype MHC2_10	0.33 ± 0.24	1.33	0.18
Genome-wide heterozygosity	-5.97 ± 8.74	-0.68	0.49
**Sex (male vs. female)**	**1.89 ± 0.31**	**6.03**	**<0.001***
**Body size**	**0.48 ± 0.12**	**3.81**	**<0.001***
Capture date	-0.005 ± 0.003	-1.48	0.14

Table S16. Associations between blood haemoglobin concentration and presence of specific MHC class I supertypes in the Eurasian coot. Bird identity and year were included as random factors in each model. The results of full and reduced model are shown. Significant predictors are marked in bold (at P < 0.05) or with asterisks (after correction for the false discovery rate).

Predictors	Estimate ± SE	z value	P
Full model			
**Intercept**	**143.23 ± 30.81**	**4.65**	**<0.001***
Supertype MHC1_1	2.26 ± 3.51	0.65	0.52
Supertype MHC1_2	1.45 ± 4.43	0.33	0.74
Supertype MHC1_3	-1.78 ± 4.29	-0.41	0.68
Supertype MHC1_4	-0.57 ± 3.97	-0.14	0.89
Supertype MHC1_5	-5.65 ± 5.12	-1.10	0.27
Supertype MHC1_6	-4.87 ± 5.09	-0.96	0.34
Supertype MHC1_7	-8.16 ± 4.35	-1.88	0.060
Supertype MHC1_8	-0.92 ± 4.03	-0.23	0.82
Supertype MHC1_9	-4.66 ± 3.04	-1.53	0.13
Supertype MHC1_10	-7.74 ± 4.70	-1.65	0.10
Supertype MHC1_11	-8.63 ± 5.42	-1.59	0.11
Supertype MHC1_12	6.15 ± 4.38	1.40	0.16
Genome-wide heterozygosity	175.91 ± 148.85	1.18	0.24
**Sex (male vs. female)**	**16.82 ± 5.55**	**3.03**	**0.002***
Body size	-1.49 ± 2.10	-0.71	0.48
**Capture date**	**-0.25 ± 0.05**	**-5.49**	**<0.001***
Reduced model			
**Intercept**	**139.65 ± 30.04**	**4.65**	**<0.001***
**Supertype MHC1_7**	**-9.40 ± 4.17**	**-2.26**	**0.024***
Supertype MHC1_9	-3.33 ± 2.94	-1.13	0.26
Supertype MHC1_10	-7.37 ± 4.65	-1.58	0.11
Supertype MHC1_11	-8.39 ± 5.45	-1.54	0.12
Genome-wide heterozygosity	197.96 ± 143.43	1.38	0.17
**Sex (male vs. female)**	**16.62 ± 5.29**	**3.14**	**<0.001***
**Body size**	**-1.45 ± 0.48**	**-0.71**	**0.020***
**Capture date**	**-0.25 ± 0.04**	**-5.66**	**<0.001***

Table S17. Associations between blood haemoglobin concentration and presence of specific MHC class II supertypes in the Eurasian coot. Bird identity and year were included as random factors in each model. The results of full and reduced model are shown. Significant predictors are marked in bold (at P < 0.05) or with asterisks (after correction for the false discovery rate).

Predictors	Estimate ± SE	z value	P
Full model			
**Intercept**	**125.55 ± 29.53**	**4.25**	**<0.001***
Supertype MHC1_1	-0.40 ± 4.29	-0.09	0.93
Supertype MHC1_2	-2.99 ± 4.15	-0.72	0.47
**Supertype MHC1_3**	**-13.52 ± 6.33**	**-2.14**	**0.033**
Supertype MHC1_4	-8.12 ± 5.07	-1.60	0.11
Supertype MHC1_5	2.93 ± 8.26	0.35	0.72
Supertype MHC1_6	-5.94 ± 4.09	-1.45	0.15
**Supertype MHC1_7**	**-8.01 ± 3.75**	**-2.13**	**0.033**
**Supertype MHC1_8**	**-16.27 ± 5.14**	**-3.16**	**0.002***
**Supertype MHC1_9**	**-9.92 ± 4.98**	**-1.99**	**0.046**
Supertype MHC1_10	-5.48 ± 4.37	-1.26	0.21
Supertype MHC1_11	-5.95 ± 3.61	-1.65	0.10
**Supertype MHC1_12**	**-13.62 ± 4.75**	**-2.87**	**0.004***
Genome-wide heterozygosity	-1.16 ± 4.95	-0.23	0.82
Sex (male vs. female)	-1.49 ± 3.54	-0.42	0.67
**Body size**	**336.46 ± 142.62**	**2.36**	**0.018**
**Capture date**	**15.35 ± 4.93**	**3.12**	**0.002***
Reduced model			
**Intercept**	**119.71 ± 28.68**	**4.17**	**<0.001***
**Supertype MHC2_3**	**-11.70 ± 5.83**	**-2.00**	**0.045**
Supertype MHC2_4	-6.75 ± 4.70	-1.44	0.15
Supertype MHC2_6	-4.80 ± 3.76	-1.28	0.20
**Supertype MHC2_7**	**-7.48 ± 3.10**	**-2.41**	**0.016***
**Supertype MHC2_8**	**-14.62 ± 4.45**	**-3.28**	**0.001***
**Supertype MHC2_9**	**-9.25 ± 3.97**	**-2.33**	**0.020***
Supertype MHC2_11	-4.89 ± 3.14	-1.56	0.12
**Supertype MHC2_12**	**-13.86 ± 3.86**	**-3.59**	**<0.001***
**Genome-wide heterozygosity**	**345.81 ± 140.84**	**2.46**	**0.010***
**Sex (male vs. female)**	**15.38 ± 4.96**	**3.10**	**0.002***
Body size	-1.67 ± 1.91	-0.87	0.38
**Capture date**	**-0.26 ± 0.04**	**-5.98**	**<0.001***

Table S18. Associations between frontal shield size and presence of specific MHC class I supertypes in the Eurasian coot. Bird identity and year were included as random factors in each model. The results of full and reduced model are shown. Significant predictors are marked in bold (at P < 0.05) or with asterisks (after correction for the false discovery rate).

Predictors	Estimate ± SE	z value	P
Full model			
Intercept	3.90 ± 2.17	1.79	0.073
Supertype MHC1_1	-0.13 ± 0.24	-0.53	0.59
Supertype MHC1_2	-0.38 ± 0.34	-1.14	0.26
Supertype MHC1_3	-0.37 ± 0.31	-1.18	0.24
Supertype MHC1_4	-0.27 ± 0.26	-1.02	0.31
Supertype MHC1_5	0.11 ± 0.36	0.31	0.76
Supertype MHC1_6	0.20 ± 0.34	0.60	0.55
Supertype MHC1_7	-0.09 ± 0.29	-0.29	0.77
Supertype MHC1_8	-0.14 ± 0.26	-0.54	0.59
Supertype MHC1_9	0.16 ± 0.21	0.77	0.44
Supertype MHC1_10	0.31 ± 0.33	0.96	0.34
Supertype MHC1_11	-0.36 ± 0.39	-0.92	0.36
Supertype MHC1_12	0.22 ± 0.31	0.71	0.48
Genome-wide heterozygosity	-11.62 ± 10.32	-1.13	0.26
**Sex (male vs. female)**	**1.53 ± 0.39**	**3.96**	**<0.001***
Body size	0.10 ± 0.14	0.66	0.51
**Capture date**	**-0.014 ± 0.004**	**-4.09**	**<0.001***

Table S19. Associations between frontal shield size and presence of specific MHC class II supertypes in the Eurasian coot. Bird identity and year were included as random factors in each model. The results of full and reduced model are shown. Significant predictors are marked in bold (at P < 0.05) or with asterisks (after correction for the false discovery rate).

Predictors	Estimate ± SE	z value	P
Full model			
Intercept	3.02 ± 2.03	1.49	0.14
Supertype MHC2_1	-0.09 ± 0.30	-0.29	0.77
Supertype MHC2_2	-0.23 ± 0.29	-0.79	0.43
Supertype MHC2_3	-0.32 ± 0.40	-0.81	0.42
Supertype MHC2_4	0.06 ± 0.35	0.17	0.86
Supertype MHC2_5	-0.58 ± 0.63	-0.92	0.36
Supertype MHC2_6	-0.13 ± 0.31	-0.44	0.66
Supertype MHC2_7	-0.22 ± 0.26	-0.85	0.40
Supertype MHC2_8	-0.24 ± 0.38	-0.63	0.53
Supertype MHC2_9	-0.24 ± 0.35	-0.68	0.50
Supertype MHC2_10	-0.15 ± 0.28	-0.52	0.60
Supertype MHC2_11	-0.40 ± 0.26	-1.57	0.12
**Supertype MHC2_12**	**-0.68 ± 0.34**	**-1.99**	**0.046**
Supertype MHC2_13	-0.35 ± 0.36	-0.98	0.32
Supertype MHC2_14	0.16 ± 0.26	0.64	0.52
Genome-wide heterozygosity	-5.99 ± 9.95	-0.6	0.55
**Sex (male vs. female)**	**1.20 ± 0.35**	**3.43**	**0.001***
Body size	0.20 ± 0.14	1.45	0.15
**Capture date**	**-0.014 ± 0.003**	**-4.18**	**<0.001***
Reduced model			
Intercept	2.81 ± 1.96	1.44	0.15
Supertype MHC2_11	-0.34 ± 0.21	-1.63	0.10
**Supertype MHC2_12**	**-0.57 ± 0.27**	**-2.11**	**0.034**
Genome-wide heterozygosity	-6.16 ± 9.43	-0.65	0.51
**Sex (male vs. female)**	**1.16 ± 0.34**	**3.42**	**0.001***
Body size	0.20 ± 0.13	1.52	0.13
**Capture date**	**-0.015 ± 0.003**	**-4.51**	**<0.001***

Table S20. Associations between laying date and presence of specific MHC class I supertypes in the Eurasian coot. Bird identity and year were included as random factors in each model. The results of full and reduced model are shown. Significant predictors are marked in bold (at P < 0.05) or with asterisks (after correction for the false discovery rate).

Predictors	Estimate ± SE	z value	P
Full model			
**Intercept**	**109.3 ± 24.76**	**4.41**	**<0.001***
Supertype MHC1_1	-0.54 ± 2.74	-0.20	0.84
Supertype MHC1_2	4.58 ± 3.64	1.26	0.21
Supertype MHC1_3	-0.79 ± 3.54	-0.22	0.82
Supertype MHC1_4	0.8 ± 3.28	0.24	0.81
Supertype MHC1_5	-0.43 ± 3.91	-0.11	0.91
**Supertype MHC1_6**	**12.62 ± 4.89**	**2.58**	**0.010**
Supertype MHC1_7	4.17 ± 3.50	1.19	0.23
Supertype MHC1_8	-1.67 ± 3.11	-0.54	0.59
Supertype MHC1_9	-2.53 ± 2.44	-1.04	0.30
**Supertype MHC1_10**	**8.33 ± 3.97**	**2.10**	**0.036**
Supertype MHC1_11	-3.46 ± 4.11	-0.84	0.40
Supertype MHC1_12	3.05 ± 3.77	0.81	0.42
Genome-wide heterozygosity	-15.22 ± 125.61	-0.12	0.90
Sex (male vs. female)	-0.64 ± 2.44	-0.26	0.79
Reduced model			
**Intercept**	**110.06 ± 22.88**	**4.81**	**<0.001***
**Supertype MHC1_6**	**12.43 ± 4.88**	**2.55**	**0.011***
**Supertype MHC1_10**	**9.85 ± 3.84**	**2.56**	**0.010***
Genome-wide heterozygosity	-5.25 ± 113.59	-0.05	0.96
Sex (male vs. female)	0.19 ± 2.37	0.08	0.94

Table S21. Associations between laying date and presence of specific MHC class II supertypes in the Eurasian coot. Bird identity and year were included as random factors in each model. The results of full and reduced model are shown. Significant predictors are marked in bold (at P < 0.05) or with asterisks (after correction for the false discovery rate).

Predictors	Estimate ± SE	z value	P
Full model			
**Intercept**	**116.75 ± 24.54**	**4.76**	**<0.001***
Supertype MHC2_1	-2.4 ± 3.58	-0.67	0.50
Supertype MHC2_2	-0.32 ± 3.38	-0.1	0.92
Supertype MHC2_3	2.69 ± 4.65	0.58	0.56
Supertype MHC2_4	-4.01 ± 4.17	-0.96	0.34
Supertype MHC2_5	-0.27 ± 8.19	-0.03	0.97
Supertype MHC2_6	-0.15 ± 3.42	-0.05	0.96
Supertype MHC2_7	2.59 ± 3.16	0.82	0.41
Supertype MHC2_8	2.36 ± 4.16	0.57	0.57
Supertype MHC2_9	3.64 ± 4.32	0.84	0.40
Supertype MHC2_10	-3.92 ± 3.69	-1.06	0.29
Supertype MHC2_11	0.05 ± 3.09	0.02	0.99
Supertype MHC2_12	-5.5 ± 4.09	-1.34	0.18
Supertype MHC2_13	0.69 ± 4.01	0.17	0.86
Supertype MHC2_14	-5.17 ± 3.01	-1.72	0.086
Genome-wide heterozygosity	-21.9 ± 121.37	-0.18	0.86
Sex (male vs. female)	0.57 ± 2.61	0.22	0.83
Reduced model			
**Intercept**	**110.84 ± 23.38**	**4.74**	**<0.001***
Supertype MHC2_14	-3.28 ± 2.68	-1.22	0.22
Genome-wide heterozygosity	2.57 ± 116.53	0.02	0.98
Sex (male vs. female)	0.49 ± 2.42	0.20	0.84

Table S22. Associations between clutch size and presence of specific MHC class I supertypes in the Eurasian coot. Bird identity and year were included as random factors in each model. The results of full and reduced model are shown. Significant predictors are marked in bold (at P < 0.05) or with asterisks (after correction for the false discovery rate).

Predictors	Estimate ± SE	z value	P
Full model			
**Intercept**	**13.06 ± 2.66**	**4.91**	**<0.001***
Supertype MHC1_1	0.23 ± 0.25	0.89	0.37
Supertype MHC1_2	0.3 ± 0.36	0.84	0.4
Supertype MHC1_3	-0.42 ± 0.33	-1.27	0.2
Supertype MHC1_4	0.33 ± 0.31	1.07	0.29
Supertype MHC1_5	0.11 ± 0.37	0.29	0.77
Supertype MHC1_6	0.12 ± 0.48	0.25	0.81
Supertype MHC1_7	0.48 ± 0.35	1.38	0.17
**Supertype MHC1_8**	**-1.18 ± 0.3**	**-3.96**	**<0.001***
Supertype MHC1_9	-0.06 ± 0.24	-0.24	0.81
Supertype MHC1_10	-0.16 ± 0.41	-0.37	0.71
Supertype MHC1_11	-0.7 ± 0.40	-1.75	0.08
Supertype MHC1_12	0.12 ± 0.37	0.32	0.75
Genome-wide heterozygosity	2.27 ± 11.54	0.20	0.84
Sex (male vs. female)	-0.01 ± 0.23	-0.05	0.96
Brood status (first vs. second)	-0.49 ± 0.98	-0.5	0.62
Brood status (renest vs. second)	0.3 ± 0.99	0.30	0.76
Laying date	**-0.05 ± 0.01**	**-6.47**	**<0.001***
Reduced model			
**Intercept**	**11.64 ± 2.49**	**4.67**	**<0.001***
**Supertype MHC1_8**	**-1.11 ± 0.30**	**-3.75**	**<0.001***
Supertype MHC1_11	-0.64 ± 0.38	-1.66	0.10
Genome-wide heterozygosity	8.86 ± 10.53	0.84	0.40
Sex (male vs. female)	0.11 ± 0.22	0.50	0.62
Brood status (first vs. second)	-0.25 ± 0.98	-0.25	0.80
Brood status (renest vs. second)	0.46 ± 1.00	0.46	0.65
Laying date	**-0.04 ± 0.01**	**-6.48**	**<0.001***

Table S23. Associations between clutch size and presence of specific MHC class II supertypes in the Eurasian coot. Bird identity and year were included as random factors in each model. The results of full and reduced model are shown. Significant predictors are marked in bold (at P < 0.05) or with asterisks (after correction for the false discovery rate).

Predictors	Estimate ± SE	z value	P
Full model			
**Intercept**	**14.33 ± 2.65**	**5.40**	**<0.001***
Supertype MHC2_1	0.07 ± 0.37	0.20	0.84
Supertype MHC2_2	0.52 ± 0.33	1.57	0.12
Supertype MHC2_3	-0.48 ± 0.46	-1.03	0.30
Supertype MHC2_4	-0.53 ± 0.45	-1.18	0.24
**Supertype MHC2_5**	**-1.94 ± 0.85**	**-2.27**	**0.023**
Supertype MHC2_6	0.58 ± 0.32	1.82	0.070
Supertype MHC2_7	-0.31 ± 0.30	-1.03	0.30
Supertype MHC2_8	0.50 ± 0.40	1.26	0.21
Supertype MHC2_9	-0.14 ± 0.42	-0.33	0.74
Supertype MHC2_10	-0.08 ± 0.36	-0.23	0.82
Supertype MHC2_11	-0.05 ± 0.31	-0.15	0.88
Supertype MHC2_12	0.47 ± 0.41	1.15	0.25
Supertype MHC2_13	0.19 ± 0.37	0.53	0.60
Supertype MHC2_14	0.24 ± 0.29	0.84	0.40
Genome-wide heterozygosity	-6.04 ± 11.28	-0.54	0.59
Sex (male vs. female)	-0.03 ± 0.25	-0.13	0.90
Brood status (first vs. second)	-0.40 ± 1.01	-0.40	0.69
Brood status (renest vs. second)	0.37 ± 1.05	0.36	0.72
Laying date	**-0.04 ± 0.01**	**-6.27**	**<0.001***
Reduced model			
**Intercept**	**13.05 ± 2.52**	**5.17**	**<0.001***
Supertype MHC2_2	0.44 ± 0.27	1.64	0.10
Supertype MHC2_5	-1.57 ± 0.81	-1.94	0.052
Supertype MHC2_6	0.49 ± 0.28	1.76	0.078
Genome-wide heterozygosity	-1.11 ± 10.59	-0.10	0.92
Sex (male vs. female)	-0.13 ± 0.24	-0.52	0.60
Brood status (first vs. second)	0.01 ± 1.00	0.01	0.99
Brood status (renest vs. second)	0.80 ± 1.03	0.77	0.44
Laying date	**-0.04 ± 0.01**	**-6.30**	**<0.001***

Table S24. Associations between hatching success and presence of specific MHC class I supertypes in the Eurasian coot. Bird identity and year were included as random factors in each model. The results of full and reduced model are shown. Significant predictors are marked in bold (at P < 0.05) or with asterisks (after correction for the false discovery rate).

Predictors	Estimate ± SE	z value	P
Full model			
Intercept	0.67 ± 4.42	0.15	0.90
Supertype MHC1_1	0.17 ± 0.42	0.41	0.68
Supertype MHC1_2	-0.24 ± 0.60	-0.41	0.68
Supertype MHC1_3	1.43 ± 0.81	1.76	0.078
Supertype MHC1_4	0.18 ± 0.55	0.33	0.75
Supertype MHC1_5	0.43 ± 0.63	0.69	0.49
Supertype MHC1_6	0.05 ± 0.91	0.06	0.96
Supertype MHC1_7	-0.07 ± 0.57	-0.13	0.90
**Supertype MHC1_8**	**-1.01 ± 0.46**	**-2.21**	**0.027**
Supertype MHC1_9	-0.39 ± 0.38	-1.02	0.31
Supertype MHC1_10	0.03 ± 0.62	0.05	0.96
Supertype MHC1_11	0.44 ± 0.68	0.65	0.52
Supertype MHC1_12	0.31 ± 0.65	0.48	0.63
Genome-wide heterozygosity	5.00 ± 19.93	0.25	0.80
Sex (male vs. female)	0.12 ± 0.38	0.32	0.75
Brood status (first vs. second)	-0.80 ± 1.25	-0.64	0.52
Brood status (renest vs. second)	0.04 ± 1.26	0.03	0.98
Laying date	0.004 ± 0.011	0.33	0.74
Reduced model			
Intercept	0.51 ± 4.31	0.12	0.91
Supertype MHC1_3	1.45 ± 0.78	1.87	0.062
**Supertype MHC1_8**	**-1.02 ± 0.44**	**-2.32**	**0.021**
Genome-wide heterozygosity	4.14 ± 18.61	0.22	0.82
Sex (male vs. female)	0.14 ± 0.36	0.40	0.69
Brood status (first vs. second)	-0.90 ± 1.22	-0.74	0.46
Brood status (renest vs. second)	-0.17 ± 1.24	-0.13	0.89
Laying date	0.01 ± 0.01	0.49	0.62

Table S25. Associations between hatching success and presence of specific MHC class II supertypes in the Eurasian coot. Bird identity and year were included as random factors in each model. The results of full and reduced model are shown. Significant predictors are marked in bold (at P < 0.05) or with asterisks (after correction for the false discovery rate).

Predictors	Estimate ± SE	z value	P
Full model			
Intercept	7.91 ± 4.68	1.69	0.091
Supertype MHC2_1	-0.31 ± 0.55	-0.56	0.57
Supertype MHC2_2	0.43 ± 0.53	0.82	0.41
Supertype MHC2_3	0.42 ± 0.74	0.57	0.57
Supertype MHC2_4	-0.24 ± 0.64	-0.37	0.71
Supertype MHC2_5	-0.23 ± 1.44	-0.16	0.87
Supertype MHC2_6	-0.43 ± 0.50	-0.85	0.40
Supertype MHC2_7	0.08 ± 0.49	0.17	0.87
Supertype MHC2_8	1.66 ± 0.86	1.92	0.055
Supertype MHC2_9	-0.07 ± 0.66	-0.11	0.91
Supertype MHC2_10	0.59 ± 0.63	0.94	0.35
Supertype MHC2_11	-0.02 ± 0.51	-0.04	0.97
Supertype MHC2_12	-0.23 ± 0.60	-0.38	0.70
Supertype MHC2_13	0.06 ± 0.64	0.09	0.93
Supertype MHC2_14	0.67 ± 0.49	1.36	0.17
Genome-wide heterozygosity	-31.35 ± 20.19	-1.55	0.12
Sex (male vs. female)	0.09 ± 0.41	0.22	0.82
Brood status (first vs. second)	-0.97 ± 1.24	-0.78	0.44
Brood status (renest vs. second)	-0.02 ± 1.245	-0.02	0.99
Laying date	0.002 ± 0.012	0.20	0.84
Reduced model			
Intercept	7.49 ± 4.42	1.70	0.090
**Supertype MHC2_8**	**1.60 ± 0.77**	**2.07**	**0.038**
Genome-wide heterozygosity	-28.84 ± 19.13	-1.51	0.13
Sex (male vs. female)	0.14 ± 0.36	0.40	0.69
Brood status (first vs. second)	-1.01 ± 1.19	-0.84	0.40
Brood status (renest vs. second)	-0.16 ± 1.22	-0.13	0.90
Laying date	0.003 ± 0.010	0.25	0.80

Table S26. Associations between breeding success and presence of specific MHC class I supertypes in the Eurasian coot. Bird identity and year were included as random factors in each model. The results of full and reduced model are shown. Significant predictors are marked in bold (at P < 0.05) or with asterisks (after correction for the false discovery rate).

Predictors	Estimate ± SE	z value	P
Full model			
Intercept	2.01 ± 1.36	1.49	0.14
Supertype MHC1_1	0.13 ± 0.12	1.06	0.29
Supertype MHC1_2	0.29 ± 0.19	1.49	0.14
Supertype MHC1_3	-0.18 ± 0.16	-1.11	0.27
Supertype MHC1_4	0.20 ± 0.15	1.35	0.18
Supertype MHC1_5	0.08 ± 0.16	0.50	0.61
Supertype MHC1_6	0.02 ± 0.26	0.08	0.94
**Supertype MHC1_7**	**-0.41 ± 0.19**	**-2.17**	**0.030**
Supertype MHC1_8	0.10 ± 0.17	0.56	0.57
Supertype MHC1_9	0.00 ± 0.11	0.01	0.99
**Supertype MHC1_10**	**0.46 ± 0.19**	**2.46**	**0.014**
Supertype MHC1_11	0.03 ± 0.17	0.18	0.86
Supertype MHC1_12	-0.09 ± 0.19	-0.51	0.61
Genome-wide heterozygosity	-0.29 ± 5.99	-0.05	0.96
Sex (male vs. female)	-0.06 ± 0.11	-0.59	0.55
Brood status (first vs. second)	-0.21 ± 0.34	0.62	0.53
Brood status (renest vs. second)	0.19 ± 0.32	0.60	0.55
Laying date	**-0.010 ± 0.004**	**-2.43**	**0.015**
Reduced model			
Intercept	0.61 ± 1.45	0.54	0.59
Supertype MHC1_7	-0.24 ± 0.16	-1.55	0.12
**Supertype MHC1_10**	**0.41 ± 0.18**	**2.32**	**0.020**
Genome-wide heterozygosity	6.79 ± 4.88	1.39	0.17
Sex (male vs. female)	0.00 ± 0.10	-0.05	0.96
Brood status (first vs. second)	0.24 ± 0.32	0.75	0.45
Brood status (renest vs. second)	0.22 ± 0.31	0.71	0.48
**Laying date**	**-0.008 ± 0.003**	**-2.24**	**0.025**

Table S27. Associations between breeding success and presence of specific MHC class II supertypes in the Eurasian coot. Bird identity and year were included as random factors in each model. The results of full and reduced model are shown. Significant predictors are marked in bold (at P < 0.05) or with asterisks (after correction for the false discovery rate).

Predictors	Estimate ± SE	z value	P
Full model			
Intercept	0.09 ± 1.28	0.07	0.95
Supertype MHC2_1	0.12 ± 0.16	0.73	0.47
Supertype MHC2_2	-0.11 ± 0.14	-0.78	0.43
Supertype MHC2_3	-0.31 ± 0.23	-1.31	0.19
Supertype MHC2_4	-0.09 ± 0.18	-0.53	0.60
Supertype MHC2_5	-0.63 ± 0.83	-0.76	0.45
**Supertype MHC2_6**	**-0.41 ± 0.18**	**-2.26**	**0.024**
Supertype MHC2_7	0.02 ± 0.14	0.17	0.87
Supertype MHC2_8	0.14 ± 0.18	0.77	0.44
Supertype MHC2_9	0.00 ± 0.18	0.01	0.99
Supertype MHC2_10	0.02 ± 0.16	0.14	0.89
Supertype MHC2_11	0.12 ± 0.14	0.87	0.38
Supertype MHC2_12	0.11 ± 0.17	0.63	0.53
Supertype MHC2_13	0.02 ± 0.18	0.13	0.90
Supertype MHC2_14	0.05 ± 0.14	0.34	0.74
Genome-wide heterozygosity	5.86 ± 5.22	1.12	0.26
Sex (male vs. female)	0.14 ± 0.12	1.20	0.23
Brood status (first vs. second)	0.35 ± 0.35	1.02	0.31
Brood status (renest vs. second)	0.22 ± 0.33	0.67	0.50
Laying date	-0.003 ± 0.004	-0.75	0.45
Reduced model			
Intercept	0.19 ± 1.14	0.16	0.87
**Supertype MHC2_6**	**-0.43 ± 0.15**	**-2.88**	**0.004***
Genome-wide heterozygosity	6.89 ± 4.79	1.44	0.15
Sex (male vs. female)	0.09 ± 0.10	0.85	0.39
Brood status (first vs. second)	0.28 ± 0.32	0.88	0.38
Brood status (renest vs. second)	0.20 ± 0.31	0.67	0.51
Laying date	-0.004 ± 0.004	-1.20	0.23

Table S28. Associations between three phenotypic traits (body mass, haemoglobin concentration and frontal shield size) and the number of MHC class I and class II supertypes in the Eurasian coot. Bird identity and year were included as random factors. Significant predictors are marked in bold

Trait	Predictors	Estimate ± SE	z value	P
Body mass (males)	**Intercept**	**32.79 ± 2.99**	**10.97**	**<0.001**
	MHC class I supertypes	0.09 ± 0.09	0.95	0.34
	**MHC class II supertypes**	**-0.47 ± 0.21**	**-2.25**	**0.024**
	Genome-wide heterozygosity	-11.67 ± 13.34	-0.87	0.38
	**Body size**	**0.66 ± 0.20**	**3.35**	**0.001**
	Capture date	-0.007 ± 0.005	-1.49	0.14
Body mass (females)	**Intercept**	**31.00 ± 2.54**	**12.21**	**<0.001**
	MHC class I supertypes	-0.16 ± 0.09	-1.78	0.076
	MHC class II supertypes	0.26 ± 0.17	1.47	0.14
	Genome-wide heterozygosity	-22.9 ± 12.5	-1.83	0.067
	**Body size**	**0.45 ± 0.19**	**2.39**	**0.017**
	Capture date	0.000 ± 0.005	-0.08	0.93
Haemoglobin concentration (males)	**Intercept**	**136.37 ± 38.61**	**3.53**	**<0.001**
	**MHC class I supertypes**	**-4.57 ± 1.17**	**-3.89**	**<0.001**
	MHC class II supertypes	-0.95 ± 2.84	-0.34	0.74
	Genome-wide heterozygosity	313.89 ± 177.23	1.77	0.077
	Body size	0.04 ± 2.31	0.02	0.99
	**Capture date**	**-0.21 ± 0.05**	**-4.31**	**<0.001**
Haemoglobin concentration (females)	**Intercept**	**156.27 ± 44.02**	**3.55**	**<0.001**
	MHC class I supertypes	0.38 ± 1.55	0.25	0.81
	**MHC class II supertypes**	**-8.60 ± 2.90**	**-2.97**	**0.003**
	Genome-wide heterozygosity	265.33 ± 213.69	1.24	0.21
	Body size	-4.22 ± 3.17	-1.33	0.18
	**Capture date**	**-0.36 ± 0.08**	**-4.59**	**<0.001**
Frontal shield size	Intercept	3.28 ± 2.03	1.62	0.11
	MHC class I supertypes	-0.03 ± 0.07	-0.40	0.69
	MHC class II supertypes	-0.20 ± 0.15	-1.37	0.17
	Genome-wide heterozygosity	-6.97 ± 9.94	-0.70	0.48
	**Sex (male vs. female)**	**1.29 ± 0.35**	**3.69**	**<0.001**
	Body size	0.17 ± 0.13	1.23	0.22
	**Capture date**	**-0.014 ± 0.003**	**-4.27**	**<0.001**

Table S29. Associations between four reproductive traits (laying date, clutch size, hatching and breeding success) and the number of MHC class I and class II supertypes in the Eurasian coot. Bird identity and year were included as random factors. Significant predictors are marked in bold

Trait	Predictors	Estimate ± SE	z value	P
Laying date	**Intercept**	**117.19 ± 23.63**	**4.96**	**<0.001**
	**MHC class I supertypes**	**1.95 ± 0.83**	**2.35**	**0.019**
	MHC class II supertypes	-3.2 ± 1.79	-1.79	0.074
	Genome-wide heterozygosity	-15.03 ± 118.27	-0.13	0.90
	Sex (male vs. female)	0.21 ± 2.46	0.08	0.93
Clutch size	**Intercept**	**12.81 ± 2.65**	**4.83**	**<0.001**
	MHC class I supertypes	-0.06 ± 0.08	-0.72	0.47
	MHC class II supertypes	0.20 ± 0.17	1.17	0.24
	Genome-wide heterozygosity	-1.27 ± 11.23	-0.11	0.91
	Sex (male vs. female)	0.01 ± 0.24	0.06	0.95
	Brood status (first vs. second)	-0.16 ± 1.04	-0.16	0.87
	Brood status (renest vs. second)	0.53 ± 1.06	0.50	0.62
	**Laying date**	**-0.04 ± 0.01**	**-5.80**	**<0.001**
Hatching success	Intercept	4.96 ± 4.26	1.16	0.24
	MHC class I supertypes	0.02 ± 0.12	0.19	0.85
	MHC class II supertypes	0.09 ± 0.26	0.35	0.73
	Genome-wide heterozygosity	-19.13 ± 18.27	-1.05	0.29
	Sex (male vs. female)	0.03 ± 0.35	0.08	0.94
	Brood status (first vs. second)	-0.75 ± 1.21	-0.62	0.54
	Brood status (renest vs. second)	-0.03 ± 1.22	-0.02	0.98
	Laying date	0.004 ± 0.011	0.37	0.71
Breeding success	Intercept	0.77 ± 1.22	0.63	0.53
	MHC class I supertypes	0.03 ± 0.04	0.82	0.41
	MHC class II supertypes	-0.02 ± 0.07	-0.27	0.79
	Genome-wide heterozygosity	7.46 ± 5.07	1.47	0.14
	Sex (male vs. female)	0.02 ± 0.11	0.21	0.83
	Brood status (first vs. second)	0.12 ± 0.34	0.34	0.73
	Brood status (renest vs. second)	0.25 ± 0.32	0.76	0.45
	**Laying date**	**-0.010 ± 0.004**	**-2.50**	**0.012**

Table S30. Association between male haemoglobin concentration and the number of MHC class I supertypes in the Eurasian coot, while accounting for the presence of specific supertypes. Bird identity and year were included as random factors. Significant predictors are marked in bold

Predictors	Estimate ± SE	z value	P
**Intercept**	**114.29 ± 39.51**	**2.89**	**0.004**
**MHC class I supertypes**	**-4.49 ± 1.81**	**-2.48**	**0.013**
Supertype MHC1_7	-1.12 ± 5.56	-0.20	0.84
Supertype MHC1_9	5.09 ± 4.55	1.12	0.26
Supertype MHC1_10	-4.90 ± 6.13	-0.80	0.42
Supertype MHC1_11	-10.07 ± 6.97	-1.44	0.15
**Genome-wide heterozygosity**	**409.90 ± 184.26**	**2.22**	**0.026**
Body size	-1.57 ± 2.43	-0.64	0.52
**Capture date**	**-0.21 ± 0.05**	**-4.29**	**<0.001**

Table S31. Association between laying date and the number of MHC class I supertypes in the Eurasian coot, while accounting for the presence of specific supertypes. Bird identity and year were included as random factors. Significant predictors are marked in bold

Predictors	Estimate ± SE	z value	P
**Intercept**	**113.28 ± 22.98**	**4.93**	**<0.001**
MHC class I supertypes	0.52 ± 0.91	0.57	0.57
Supertype MHC1_6	-2.81 ± 1.72	-1.63	0.10
**Supertype MHC1_10**	**11.06 ± 5.05**	**2.19**	**0.029**
**Genome-wide heterozygosity**	**9.19 ± 4.17**	**2.21**	**0.027**
Sex (male vs. female)	10.53 ± 114.75	0.09	0.93

Table S32. Association between male body mass and the number of MHC class II supertypes in the Eurasian coot, while accounting for the presence of specific supertypes. Bird identity and year were included as random factors. Significant predictors are marked in bold

Predictors	Estimate ± SE	z value	P
**Intercept**	**30.51 ± 2.79**	**10.95**	**<0.001**
**MHC class II supertypes**	**-0.44 ± 0.20**	**-2.19**	**0.029**
**Supertype MHC2_1**	**-0.87 ± 0.29**	**-2.98**	**0.003**
Supertype MHC2_3	-0.32 ± 0.98	-0.32	0.75
Supertype MHC2_7	-0.14 ± 0.27	-0.51	0.61
Supertype MHC2_8	-0.45 ± 0.42	-1.07	0.28
**Supertype MHC2_10**	**0.69 ± 0.31**	**2.21**	**0.027**
Genome-wide heterozygosity	2.18 ± 12.80	0.17	0.86
**Body size**	**0.51 ± 0.18**	**2.92**	**0.004**
Capture date	-0.007 ± 0.004	-1.60	0.11

Table S33. Association between female haemoglobin concentration and the number of MHC class II supertypes in the Eurasian coot, while accounting for the presence of specific supertypes. Bird identity and year were included as random factors. Significant predictors are marked in bold

Predictors	Estimate ± SE	z value	P
**Intercept**	**127.23 ± 42.88**	**2.97**	**0.003**
**MHC class II supertypes**	**-6.77 ± 2.82**	**-2.40**	**0.016**
Supertype MHC2_3	-11.28 ± 6.38	-1.77	0.077
Supertype MHC2_7	-7.54 ± 4.51	-1.67	0.095
Supertype MHC2_8	-11.53 ± 5.99	-1.93	0.054
Supertype MHC2_9	-1.65 ± 6.60	-0.25	0.80
Supertype MHC2_12	-9.12 ± 5.36	-1.70	0.089
**Genome-wide heterozygosity**	**424.27 ± 208.90**	**2.03**	**0.042**
Body size	-3.47 ± 3.14	-1.11	0.27
**Capture date**	**-0.35 ± 0.08**	**-4.59**	**<0.001**

Fig. S1. Bayesian Information Criterion (BIC) values for different numbers of clusters of MHC class I (A) and MHC class II (B) alleles in the Eurasian coot.

A

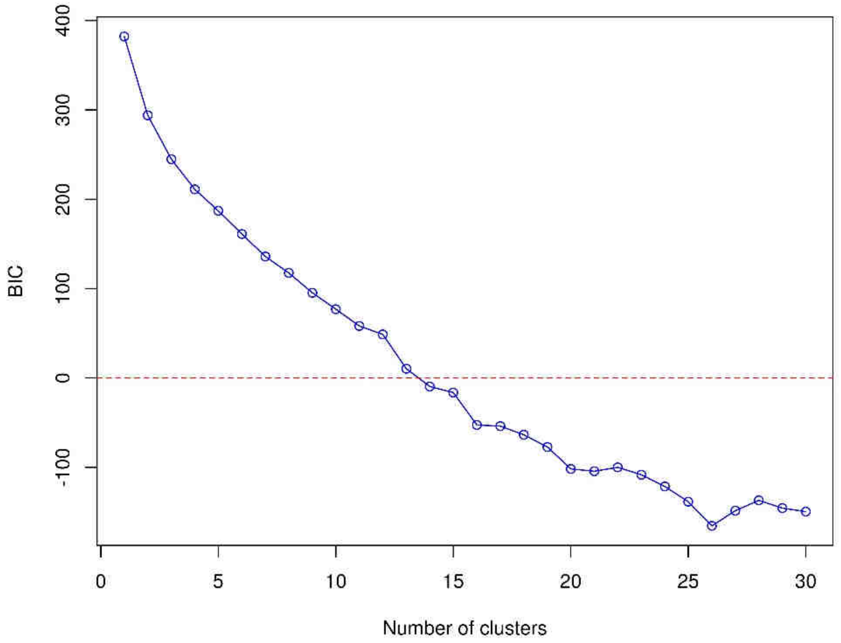

B

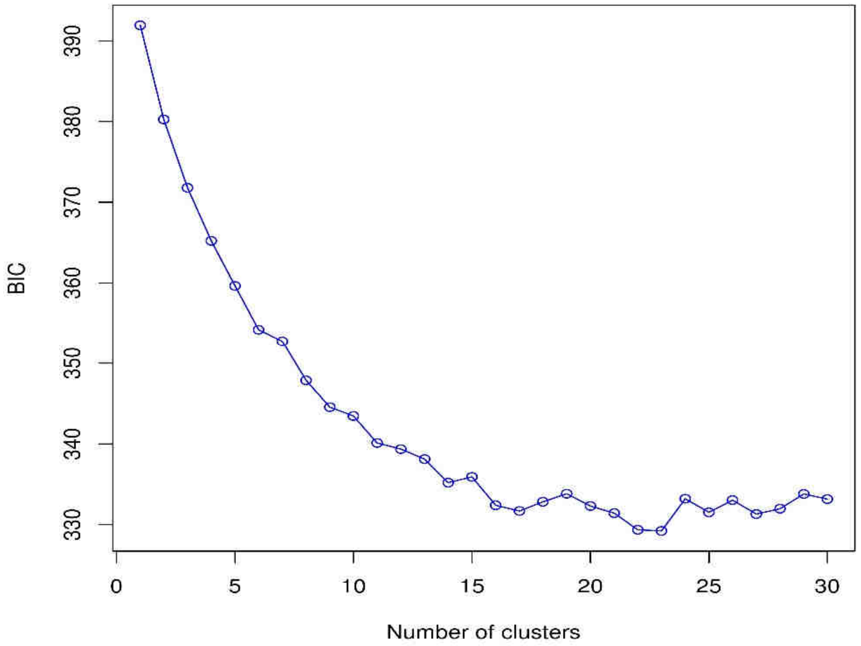

Supplement: Supplementary file 1 — Data S1 [file JANE-91-1707-s001.pdf]
